# Medication adherence and influencing factors in children and adolescents with attention deficit hyperactivity disorder (ADHD): a systematic review and meta-analysis

**DOI:** 10.3389/fphar.2026.1819206

**Published:** 2026-07-23

**Authors:** Jianing Liu, Bingyao Kang, Weiqi Deng, Zilong Hao, Chunsong Yang, Lingli Zhang

**Affiliations:** 1 Department of Pharmacy/Evidence-Based Pharmacy Center, West China Second University Hospital, Sichuan University, Children’s Medicine Key Laboratory of Sichuan Province, Chengdu, China; 2 NMPA Key Laboratory for Technical Research on Drug Products in Vitro and in Vivo Correlation, Chengdu, China; 3 Key Laboratory of Birth Defects and Related Diseases of Women and Children, Sichuan University, Ministry of Education, Chengdu, China; 4 West China School of Medicine, Sichuan University, Chengdu, China; 5 Chinese Evidence-based Medicine Center, West China Hospital, Sichuan University, Chengdu, China; 6 Department of Pediatric Clinic, West China Second University Hospital, Sichuan University, Chengdu, China; 7 West China School of Pharmacy, Sichuan University, Chengdu, China; 8 Department of Neurology, West China Hospital, Sichuan University, Chengdu, China; 9 Medical Big Data Center, Sichuan University, Chengdu, China

**Keywords:** ADHD, attention deficit hyperactivity disorder, children and adolescents, medication adherence, systematic review

## Abstract

**Background:**

Medication non-adherence is widely occurring in children and adolescents with ADHD, which may lead to adverse consequences. However, the pooled adherence rate and influencing factors are inconsistent in different studies.

**Methods:**

We searched PubMed, Embase (Ovid), Cochrane Library, and four Chinese Databases from inception to 20 February 2024. All types of studies were included. Two researchers independently extracted data and assessed the risk of bias based on ROBINS-I, NOS, and AHRQ. A random-effects meta-analysis was conducted to pool the prevalence estimates and factors that can be combined.

**Results:**

After screening 2,360 publications, 63 studies were finally included, involving 450,759 participants. The general quality of the included studies was moderate to high quality. Adherence rates were reported in 50 studies, and a pooled adherence rate was 43.6% (95% Confidence Interval [CI] 39.0%–48.3%). The sensitivity analysis shows robust study results. However, both the primary analysis and the sensitivity analysis indicated high heterogeneity. The results of Meta-analysis identified five factors influencing adherence, of which female (Odds Ratio [OR] = 1.07, 95%CI:1.03–1.11), fewer medication doses (OR = 0.45, 95%CI:0.27–0.75), parental agreement with the medication plan (OR = 1.87, 95%CI:1.16–3.02), single-child families (OR = 1.80, 95%CI:1.29–2.52), and medication education for parents (OR = 6.34, 95%CI:3.97–10.12) can improve medication adherence. Besides, we also identified seven factors influencing adherence by qualitative analysis.

**Conclusion:**

Medication adherence is challenging for children and adolescents with ADHD, moreover, the meta-analysis of adherence rates showed high heterogeneity. We identified five modifiable factors by meta-analysis that are related to medication adherence, while other factors require further research.

**Systematic Review Registration:**

identifier CRD42024514310.

## 1 Introduction

Attention Deficit/Hyperactivity Disorder (ADHD) is a neurodevelopmental disorder with inattentive, disorganized, hyperactive, and impulsive behaviors as its main clinical manifestations (Coghill et al., 2023). An umbrella review of 13 meta-analyses, including 588 studies with 3,277,590 patients from Africa, China, India, the US, Canada, Spain and so on, found a pooled prevalence of ADHD of 8.0% (95%CI 6–10%); however, ranging from different countries (Ayano et al., 2023). Comorbidity of psychiatric disorders is considered common in ADHD, the most common comorbidity was Oppositional Defiant Disorder (ODD, 34.7%), followed by Anxiety Disorders (18.4%), Specific phobias (11.0%), Enuresis (10.8%), and Conduct Disorder (CD) (10.7%) (Njardvik et al., 2025). In Australia, a study revealed a total cost of AUD 20.57 billion in 2019, indicating high costs and a heavy burden at the individual, family, and societal level (Australian ADHD Professionals Association, 2019; Njardvik et al., 2025). Medication treatment is one of the most common treatments for ADHD, which is categorized into stimulants (methylphenidate, amphetamines, etc.) and non-stimulants (atomoxetine, guanfacine, clonidine, etc.) (Mechler et al., 2022; Posner et al., 2020; Wolraich et al., 2019). The treatment effects will be influenced by medication adherence.

Medication adherence, which is defined as the extent to which patients take their medications as prescribed by their healthcare provider (Stirratt et al., 2018), is very improtant for medication treatment. There have been a lot of medication adherence measurements. However, no single measurement strategy has been deemed optimal, and it still remains global challenge because of the absence of gold standard methods for adherence measurement (Mason et al., 2022; WHO, 2003). Medication adherence measurements could be categorized into subjective measurements (e.g., Medication Adherence Rating Scale, MARS), and objective measurements (e.g., medication possession ratio, MPR; proportion of days covered, PDC, etc.) (Osterberg and Blaschke, 2005). Self-management during medication treatment significantly influences medication adherence. However, children and adolescents are often faced with shared management among multiple participants, with the primary role switching from parent-directed in childhood to self-directed in adolescence during the development (Kieckhefer et al., 2009; Lozano and Houtrow, 2018). Thus, it will be challenging to achieve good medication adherence in children and adolescents.

For children and adolescents with other diseases, medication non-adherence may be associated with more emergency department visits, increased risk for hospitalization, increased healthcare use, and need for treatment escalation (Carmody et al., 2019; McGrady and Hommel, 2013). In children and adolescents with ADHD, a cohort study found discontinuation of medication in children with ADHD will lead to poor treatment outcomes whether medication is resumed or not, and children who stopped medication (162/166, 97.5%) had at least one impairment at the follow-up assessment time point after the last reported stop (Brinkman et al., 2018a). Another study found that good adherence may be protective against the risk of co-morbidities, and ADHD patients with good adherence have a significantly lower risk of oppositional defiant disorder (ODD) and conduct disorder (CD) (Wang et al., 2018). Therefore, it is necessary to understand the current status and explore the risk factors of medication adherence in children and adolescents with ADHD.

There have been some studies reporting the medication adherence of children and adolescents with ADHD. A systematic review reported the pooled medication adherence rate of 0.57 (Number of studies = 2) in children and adolescents with ADHD, and the qualitative analysis indicated lower medication persistence in children and adolescents, with a mean duration of 136 days over the 1-year follow-up compared to 230 days in adults (Gajria et al., 2014). In children with ADHD, medication non-adherence widely occurred, with some patients stopping medication completely or periodically (Brinkman et al., 2018b). In children and adolescents with ADHD, early medication discontinuation is common; only 65% of children and 47% of adolescents remained on treatment within 1 year of initiation (Brikell et al., 2024). A recent study in Saudi Arabia reported a high medication adherence rate of 89.77% in 6 to 8-year-old children with ADHD (Alsubaie et al., 2024). However, a study in Malaysia reported a medication adherence rate of 53.1% in children and adolescents. Given that the decade-old systematic review only included two child-specific studies and the highly variable adherence rates in recent pediatric studies (Cheung et al., 2021; Earla et al., 2022; Ishizuya et al., 2021; Nayak et al., 2021), a new systematic review is needed to clarify current adherence rates.

According to former studies, the risk factors of medication adherence can be divided into patient-related (e.g., age, gender, patient mood), disease-related (e.g., comorbidity), medication-related (e.g., Duration of therapy, formulation of medication), caregiver-related (e.g., age of caregiver), and environment-related (e.g., Socioeconomic status) (Gast and Mathes, 2019; Nizet et al., 2022; Religioni et al., 2025; Yang et al., 2019). Although existing studies have found that the severity of ADHD, ODD/CD, anxiety symptoms, age, patient mood, and resistance to medication may be influential factors in adherence in ADHD patients (Emilsson et al., 2020), there is still need to summarize (Yang et al., 2019).

In this study, we conduct a systematic review to evaluate the medication adherence of children and adolescents with ADHD and identify factors affecting medication adherence in these patients.

## 2 Methods

This study is reported according to the Preferred Reporting Items for Systematic Reviews and Meta-Analyses (PRISMA) statement (Page et al., 2021). The protocol for the study was registered with PROSPERO (CRD42024514310).

### 2.1 Inclusion and exclusion criteria

#### 2.1.1 Inclusion criteria

Study Design: We included randomized controlled trials (RCTs), non-randomized studies of interventions (NRSIs), cohort studies, case-control studies, and cross-sectional studies in this study.

Participants: Children and adolescents (≤18 years old) with ADHD were included. The diagnostic criteria include the Diagnostic and Statistical Manual of Mental Disorders (DSM) (APA American Psychiatric Association, 1987; APA American Psychiatric Association, 1994; APA American Psychiatric Association, 2000), the International Classification of Diseases (ICD) (WHO, 2025), and the Diagnosis and Treatment Recommendations for Attention Deficit Hyperactivity Disorder in Children (a recommendation in Chinese) (The Editorial Board of Chinese Journal of Pediatrics, Chinese Pediatric Neurology Society and Healthcare, 2006).

Interventions/Exposure: The intervention group should be treated with ADHD therapeutic drugs, including stimulants and non-stimulants. Stimulants include Methylphenidate-based Preparations (e.g., Methylphenidate) and Amphetamine-based Preparations (e.g., Amphetamine). Non-stimulants include selective norepinephrine reuptake inhibitors (e.g., atomoxetine), alpha-2 adrenergic agonists (e.g., guanfacine and clonidine), tricyclic antidepressants (e.g., imipramine), and bupropion, and so on (Wolraich et al., 2019).

Outcome: The primary outcome is medication adherence. And the methods to evaluate medication adherence including objective methods and subjective methods. The former includes the medication possession ratio (MPR) and proportion of days covered (PDC), and the later includes the Medication Adherence Rating Scale (MARS) and self-defined adherence measurement methods (Self-defined methods refers to measurement methods which were defined by the authors of the included studies without reporting whether the methods were validated, more details in Supplementary Table S1). Secondary outcomes include factors that have an impact on medication adherence.

#### 2.1.2 Exclusion criteria

Studies were excluded: 1) Duplication; 2) Literature without full text; 3) Literature in non-English or Chinese; 4) data on children could not be extracted; 5) studies exploring the relationship between non-pharmacologic treatments and adherence.

### 2.2 Searches

We searched PubMed, Embase (Ovid), Cochrane Library, CBM, CNKI, Wanfang, and VIP Database from inception to 20 February 2024. The search terms were (Attention Deficit Disorder with Hyperactivity OR ADHD) AND (Medication Adherence OR adherence) AND (Adolescent OR Child OR Infant), and the search strategy was adjusted specifically for each database. In addition, we included additional studies by reading references. The detailed search strategy is shown in the Supplementary Appendix 1.

### 2.3 Data extraction

Two authors extracted data independently using a predefined Excel Data Extraction Form. Any disagreement is resolved by discussion. The items included basic information (author, publication year, region, study type, diagnostic criteria, source of patient), baseline characteristics of the study population (sample size, age, co-morbidities), interventions (intervention details, follow-up period, and adherence measurement methods, and risk factors of medication adherence), elements of risk of bias evaluation, outcomes (drug adherence rate and factors which influence medication adherence). When extracting adherence data, if a study can provide separate adherence information for different drugs and different follow-up times, we extracted these data separately for subsequent analysis. Therefore, a study can provide multiple adherence rate information.

### 2.4 Quality assessment

To evaluate the risk of bias for each article, we used different evaluation manual tools based on the type of included studies. For RCTs, we used the Cochrane Risk of Bias (RoB) (Higgins et al., 2011); for non-RCTs, we used Risk Of Bias In Non-randomized Studies of Interventions (ROBINS-I) (Sterne et al., 2016); for cohort studies and case-control studies we used the Newcastle-Ottawa Scale (NOS) (Cook and Reed, 2015); and for cross-sectional studies, we used the Agency for Healthcare Research and Quality (AHRQ) Cross-sectional Evaluation Quality Assessment Tool (Yang et al., 2019).

### 2.5 Data analysis

For medication adherence, the pooled prevalence rate and 95% confidence interval (CI) were calculated using Stata 17.0 (Stata Statistical Software: College Station, TX: Stata Corp LP). The degree of heterogeneity was determined by *I* ^*2*^, and the statistical analysis was performed using the random-effects model regardless of heterogeneity. The descriptive analysis was used to describe the results of studies if the data cannot be pooled. Interventional studies, such as RCTs and non-RCTs, typically evaluate the impact of interventions on adherence. And the intervention group will be influenced by the interventions, which do not reflect patients’ real-world medication adherence, whereas the control group represents actual adherence patterns. Therefore, we exclusively included adherence outcomes from control groups (details of the types of interventions of these RCTs and non-RCTs are shown in Supplementary Table S1). We excluded interventions as adherence factors in subsequent analyses to ensure objectivity. Pre-planned subgroup analyses were conducted based on study type, drugs and adherence measurement methods, of which were divided into study type (cohort, case-control, cross-section, RCT, NRSI), formulation (to identify the impact of medication formulations, such as IR, SR, ER, and OROS), drugs (to identify the impact of specific medications), drug types (to identify the impact of different drug types such as sitimulants and non-sitimulants), and adherence measurement methods (MPR, PDC, and so on), and. type of adherence measurement methods (subjective, or objective).

In the preregistered protocol, we preplanned to conducted subgroup analysis based on the study type, drugs, and adherence measurement methods. However, as subgroup analysis failed to address the problem of high heterogeneity, we conducted a *post hoc* subgroup analysis based on country, source of participants (clinical department, or electronic health records, EHR), diagnostic criteria (ICD, DSM, etc.), time for follow-up (12m, 24m, and so on). Besides, a meta-regression was conducted to explore the potential reasons for heterogeneity based on Stata 17.0. The covariates included in the meta-regression were age, study type, country, diagnostic criteria, source, formulation, drugs, drug type, follow-up time, and adherence.

For risk factors of medication adherence, we categorized the extracted factors into different categories based on thematic analysis.

As we only included studies in English and Chinese, we assessed publication bias by funnel plot, Egger’s test and the Trim-and-filled method, which is a nonparametric method for estimating the number of missing studies that might exist in a meta-analysis and the effect that these studies might have had on its outcome (Duval and Tweedie, 2000).

We conducted four types of sensitivity analyses to determine the robustness. In the first analyses, We excluded low-quality studies. Besides, we identified the top 5 high heterogeneity studies by the Baujat plot (R 4.3.3.), excluded these studies, and re-conducted meta-analysis, presented funnel plots and Trim-and-filled method. In the third type of sensitivity analysis, we excluded all studies that used newly defined adherence measurement methods or did not report detailed adherence measurement methods. In the third type of sensitivity analysis, we excluded all studies that used newly defined adherence measurement methods because these methods may be unvalidated. We also excluded studies that did not report detailed adherence measurement methods because we lacked relevant information to evaluate the scientific quality of those methods. The remaining studies were grouped according to whether the adherence measurement was subjective or objective, and subgroup analyses were conducted, with adherence rates reported separately. In the forth type of sensitivity analysis, we applied stricter inclusion and exclusion criteria: in addition to excluding studies based on the adherence measurement methods, we also excluded studies that did not clearly report diagnostic criteria, specific medications, or follow-up time.

## 3 Results

### 3.1 Literature research

**FIGURE 1:**
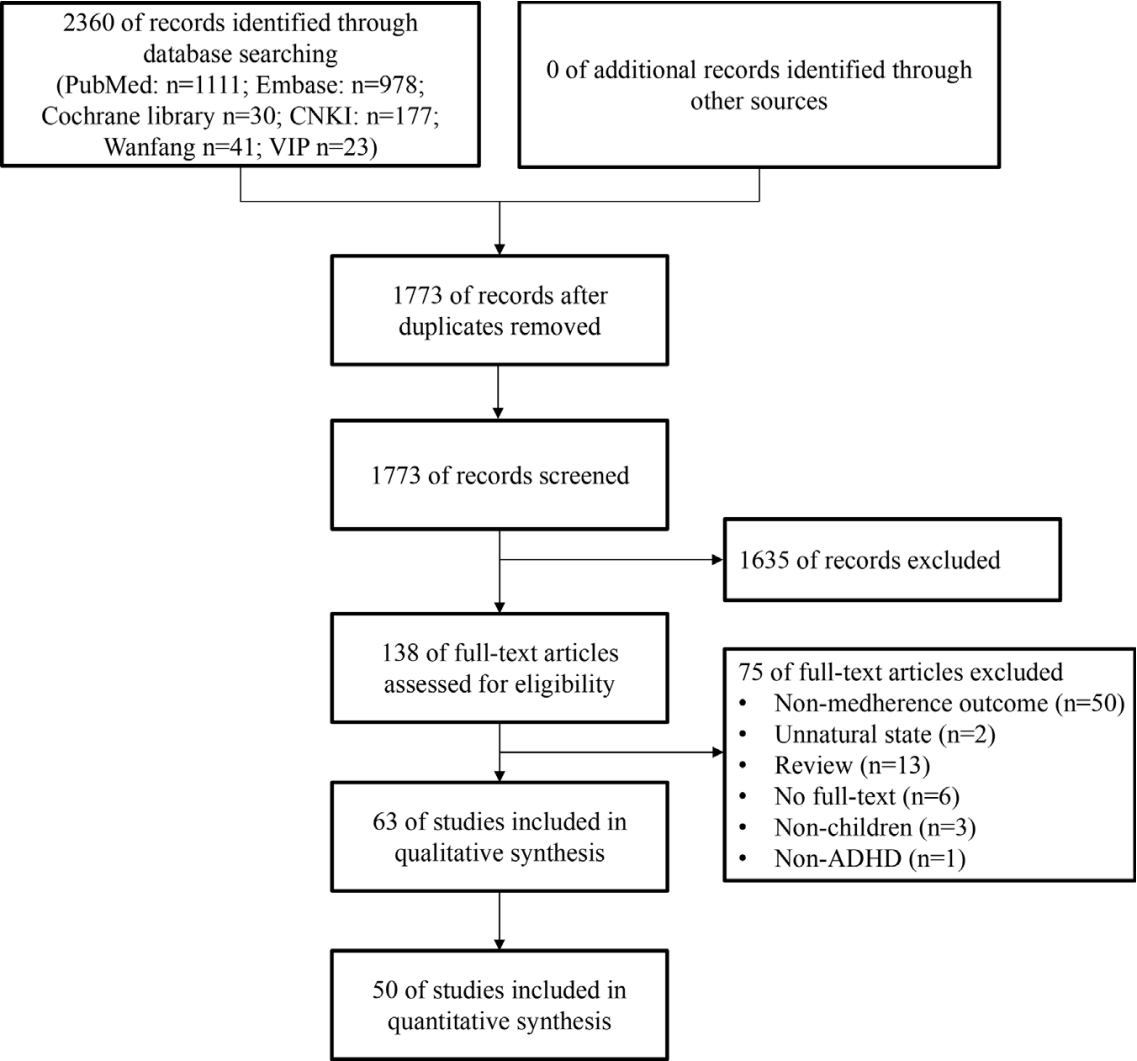
Literature screening flowchart.

The initial search identified a total of 2,360 studies. After literature screening, 63 studies were finally included for qualitative analysis. Thirteen studies were included only for qualitative synthesis because the adherence rate cannot be calculated, and 50 studies were included in the quantitative analysis (Figure 1).

### 3.2 Study characteristics of included studies

**FIGURE 2:**
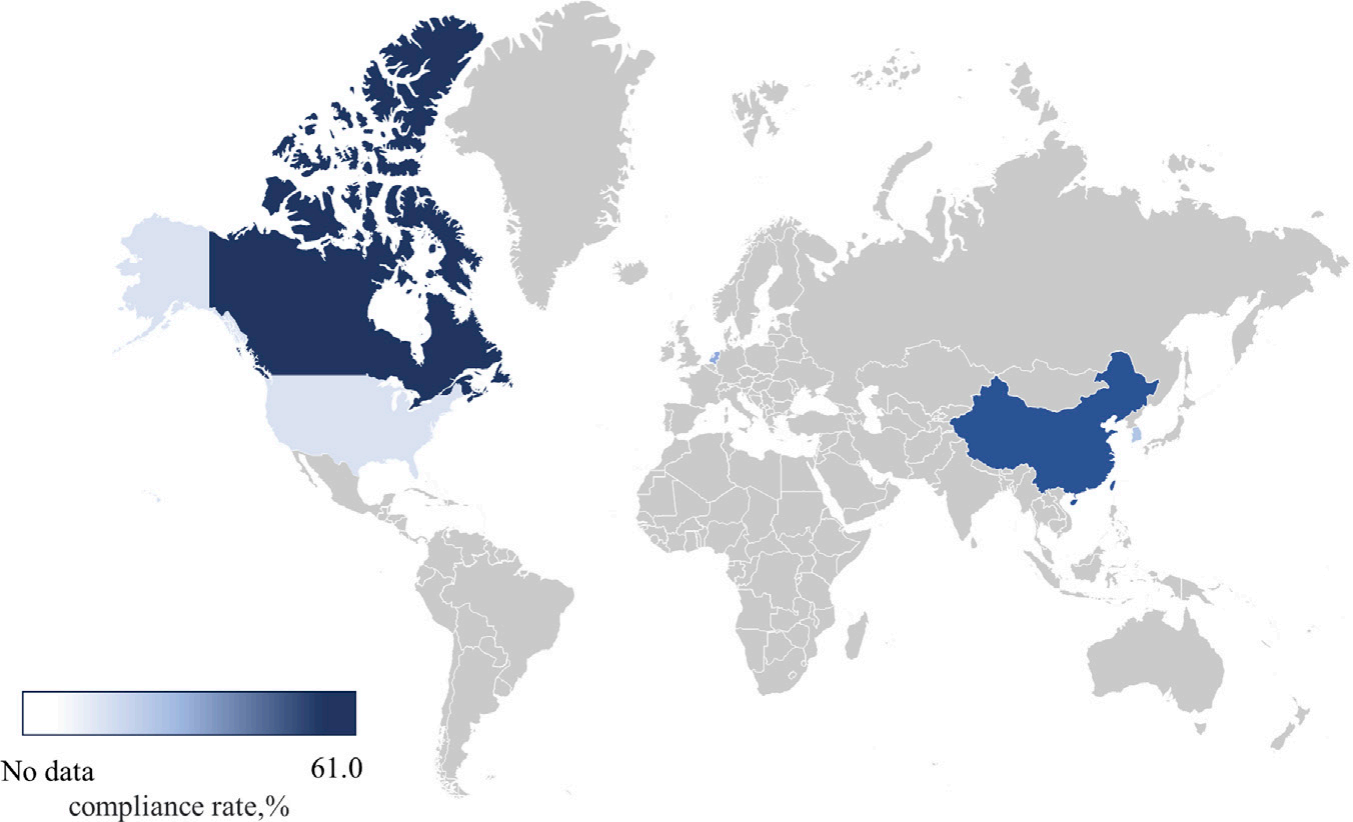
The distribution of studies on adherence rate.

For study type, 14 articles were RCTs (22.2%), 23 articles were cohort studies (36.5%), 24 articles were cross-sectional studies (38.1%), and 2 articles were NRSIs (3.2%). Of them, 34 studies were conducted in Asia, while 20 were in North America, and 9 in Europe (Figure 2).

The sample size ranged from 18 to 53,907 (median 204, Interquartile Range [IQR] 496). The ages ranged from 5 to 18 years. The patients included were mainly male (50.7%∼91.2%). As shown in Table 1, the inclusion of patients mainly comes from the clinical department (42/63, 66.7%) and EHR (11/63, 17.5%). In terms of diagnostic criteria, DSM-IV was the most commonly used diagnostic criteria (18/63, 28.6%). In terms of intervention measures, 54 studies reported medication information, with MPH being the main treatment drug (46/54, 85.2%). Most of the included studies (33/63, 52.38%) used newly self-defined adherence measurement methods (Supplementary Table S2). 45 articles reported the time for evaluating adherence outcomes, with follow-up periods at 12 months (12/45, 26.7%), 6 months (9/45, 20.0%), and 3 months (6/45, 13.3%) (details of each study were shown in Supplementary Table S3).

**TABLE 1:** Study characteristics of included studies.

Category	Group	N of study	Category	Group	N of study
Evaluation time	NI	18	Diagnostic criteria	DSM-IV	18
	12 M	12		NI	16
	6 M	9		DSM-V	6
	3 M	7		ICD-10	5
	36 M	4		ICD-9	4
	12 W	2		DSM-III	3
	1 M	2		DSM-III-R	2
	Others	12		Others	9
Adherence	Newly	33	Source	CD	42
	MPR	12		EHR	12
	MARS or morsiky	7		Schools	5
	Other	6		NI	2
	NI	3		Trail	1
	PDC	2		Mixed	1

### 3.3 Results of quality assessment and risk of bias

The results of risk of bias of the inclued studies were shown in Table 2. Most of the cohort studies and cross-sectional studies were in low risk of bias, while RCT and NRSI were mainly of high risk of bias. More details of risk of bias of each study were shown in Supplementary Tables S4–S7, and Supplementary Figures S1, S2.

**TABLE 2:** Results of risk of bias of included studies.

Study type	Risk of bias	N of study
Cohort studies	Low	16
	Medium	7
Cross-sectional studies	Low	12
	Medium	12
RCT	High	9
	Medium	5
NRSI	High	2

### 3.4 Results of medication adherence

#### 3.4.1 Medication adherence of quantity synthesis

A total of 50 studies were included for quantitative analysis, and the same study may have provided multiple data points due to differences in follow-up time and medication type. The adherence rate ranged from 3.39% to 100%, and the meta-analysis showed an overall adherence rate of 43.6% (95%CI 39.0%–48.3%, *I* ^*2*^ = 99.85%) (Supplementary Figure S5).

Subgroup analysis was conducted based on the study type of included studies, country, source, diagnostic criteria, time for follow-up, and age, and the results of subgroup analyses are shown in Table 3. Furthermore, a meta-regression was conducted to explore the potential reasons for heterogeneity. The covariates included in the meta-regression were age, study type, country, diagnostic criteria, source, formulation, drugs, drug type, follow-up time, and adherence. The results of the univariate analysis indicated that study type, country, diagnostic criteria, source, formulation, and drugs might be the causes of heterogeneity. The results of multivariate analysis showed that study type, country, diagnostic criteria, source, formulation, and drug type could be the potential sources of heterogeneity (Table 4).

**TABLE 3:** Subgroup analysis in quantitative analysis of medication adherence rates.

Subgroup	N of study	N of patients	Results of heterogeneity		Effect model	Results of meta analysis	
			*I* ^2^	*P*		Adherence rate (%)	95%CI
Study type							
Intervention	12	4,713	97.72%	<0.01^*^	Random	56.9	45.3–68.2
Cross-section	21	3,563	96.33%	<0.01	Random	51.2	42.2–60.2
Cohort	16	319,814	99.91%	<0.01	Random	37.5	31.9–43.3
Country							
Canada	4	2,413	96.72%	<0.01	Random	61.0	43.4–77.2
China	20	7,746	97.15%	<0.01	Random	56.8	49.6–63.8
Netherlands	3	5,327	91.70%	<0.01	Random	41.1	33.3–49.1
Korean	4	140,545	99.98%	<0.01	Random	28.2	12.5–47.2
America	6	168,395	99.61%	<0.01	Random	19.5	16.5–22.7
Diagnosis							
Other	2	112	71.15%	<0.01	Random	64.7	50.6–77.6
DSM-III-R	2	505	85.71%	<0.01	Random	54.0	42.4–65.5
DSM-IV	15	4,342	97.20%	<0.01	Random	51.9	42.7–61.1
DSM-V	5	2,987	94.58%	<0.01	Random	49.8	34.6–64.9
NI	12	71,867	99.42%	<0.01	Random	44.3	39.1–49.6
ICD-10	4	141,859	99.98%	<0.01	Random	28.6	13.0–47.4
ICD-9	1	101,616	99.65%	<0.01	Random	13.8	10.4–17.6
Source							
CD	31	5,222	96.63%	<0.01	Random	57.8	51.6–63.8
EHR	11	213,408	99.94%	<0.01	Random	21.3	15.7–27.4
Formulation							
SR MPH	2	632	89.76%	<0.01	Random	83.8	73.8–91.9
MPH + ATX	6	1,051	93.78%	<0.01	Random	57.7	44.1–70.7
Stimulants	5	1,633	93.66%	<0.01	Random	55.6	45.1–65.9
IR MPH	10	11,946	99.44%	<0.01	Random	52.0	37.3–66.5
ER MPH	5	8,256	98.82%	<0.01	Random	47.8	34.1–61.7
OROS MPH	8	45,320	99.43%	<0.01	Random	43.8	36.8–50.9
NI	4	6,003	99.34%	<0.01	Random	43.6	25.4–62.7
Mixed	9	133,384	99.97%	<0.01	Random	39.2	22.3–57.6
MPH	7	37,834	99.47%	<0.01	Random	34.6	27.2–42.4
ATX	7	19,403	98.34%	<0.01	Random	24.1	18.7–29.9
AMPH	3	43,285	99.18%	<0.01	Random	12.7	9.0–16.9
Drugs							
Stimulants	5	1,633	93.66%	<0.01	Random	55.6	45.1–65.9
MPH	24	85,036	99.51%	<0.01	Random	50.7	45.9–55.4
Mixed	15	134,435	99.95%	<0.01	Random	46.4	32.3–60.8
NI	4	6,003	99.34%	<0.01	Random	43.6	25.4–62.7
ATX	7	19,403	98.34%	<0.01	Random	24.1	18.7–29.9
AMPH	3	43,285	99.18%	<0.01	Random	12.7	9.0–16.9
Drug type							
Mixed	15	134,435	99.95%	<0.01	Random	46.4	32.3–60.8
Stimulants	29	168,249	99.47%	<0.01	Random	44.5	41.0–48.1
NI	4	6,003	99.34%	<0.01	Random	43.6	25.4–62.7
Non-stimulants	7	19,403	98.34%	<0.01	Random	24.1	18.7–29.9
Follow time							
1 M	4	597	90.34%	<0.01	Random	89.1	78.2–96.7
3 M	7	899	96.29%	<0.01	Random	66.7	48.6–82.6
6 M	12	19,844	96.83%	<0.01	Random	43.8	37.4–50.2
NI	13	133,549	99.93%	<0.01	Random	38.1	26.7–50.2
24 M	4	15,539	98.20%	<0.01	Random	37.1	17.1–59.7
36 M	5	25,752	99.60%	<0.01	Random	35.8	24.8–47.7
12 M	11	125,517	99.67%	<0.01	Random	31.7	27.1–36.6
Adherence							
Other	5	850	79.81%	<0.01	Random	74.5	66.1–82.1
New	24	6,584	96.47%	<0.01	Random	58.0	51.4–64.6
NI	3	3,366	97.87%	<0.01	Random	55.9	38.8–72.3
MARS	8	823	98.01%	<0.01	Random	49.1	25.0–73.5
MPR	9	211,768	99.95%	<0.01	Random	28.4	20.0–37.5
PDC	2	104,699	99.62%	<0.01	Random	13.9	10.7–17.5
Adherence type							
Subjective	8	823	98.01%	<0.01	Random	49.1	25.0–73.5
Objective	10	316,467	99.94%	<0.01	Random	22.9	17.2–29.2

* *P*-values less than 0.01 are reported as <0.01 for clarity.

Study type. Cohort: cohort study, Cross-section: cross-section study, RCT: randomised controlled study, NRSI: non-randomised studies of the effects of interventions. Source: the source of participants, including clinical department, scool, and electronic health records. CD: clinical department, EHR: electronic health records. Formulation: this category represents the impact of medication formulations, such as IR, SR, ER, and OROS; if a specific formulation was not reported, only the drug name itself was retained. Drugs: MPH: methylphenidate, ATX:Atomoxetine, AMPH:Amphetamine, Mixed: all type of drugs. In this category lists the specific medications (e.g., AMPH, MPH, LDX), not the broader drug classes; if the literature did not specify a drug name and only indicated the drug class, that class name was used for presentation. Drug types: this category classifies and presents the specific medications based on their overarching type as either a Stimulant or a Non-stimulant. Adherence: MARS:Medication Adherence Rating Scale, MPR:medication possession ratio, PDC: proportion of days covered, Newly: Newly defined methods. Adherence type: Subjectvie: MARS, objective: MPR and PDC.

**TABLE 4:** Results of meta-regression.

Covariate	Univariate analysis			Multivariate analysis		
	β	95%CI	*P*	β	95%CI	*P*
Studytype	0.07	0.02, 0.13	0.004	0.05	0.009.0.26	0.000^*^
Country	0.01	0.004, 0.022	0.004	0.07	0.03.0.11	0.000^*^
Diagnosis	−0.01	−0.022, −0.002	0.023	0.009	0.0008.0.02	0.033^*^
Source	−0.09	−0.12, −0.06	0.000	−0.07	−0.09,-0.04	0.000^*^
Age	0.0002	−0.01, 0.02	0.973	−0.01	−0.02.0.002	0.107
Formulation	0.03	0.02, 0.04	0.000	0.02	0.009.0.03	0.001^*^
Drugs	0.07	0.04, 0.09	0.000	0.03	−0.004.0.06	0.089
Drugtypes	−0.001	−0.03, 0.03	0.941	0.03	0.007.0.06	0.012^*^
Followtime	0.0009	−0.005, 0.006	0.784	−0.002	−0.009.0.004	0.484
Adherence	−0.006	−0.03, 0.02	0.610	0.01	−0.01.0.04	0.363

Formulation: this category represents the impact of medication formulations, such as IR, SR, ER, and OROS; if a specific formulation was not reported, only the drug name itself was retained. Drugs: this category lists the specific medications (e.g., AMPH, MPH, LDX), not the broader drug classes; if the literature did not specify a drug name and only indicated the drug class, that class name was used for presentation. Drug types: this category classifies and presents the specific medications based on their overarching type as either a Stimulant or a Non-stimulant.

#### 3.4.2 Medication adherence of qualitative synthesis

Considering the high heterogeneity of medication adherence, we also conducted a qualitative synthesis of the 50 studies. For the study type, the median and IQR of medication adherence in cohort, cross-sectional, and interventional studies are 28.02% (16.38%–66.54%), 52.63% (38.44%–62.13%), and 59.00% (36.23%–76.30%). More details were shown in Supplementary Table S8.

Thirteen articles cannot be quantitatively analyzed, so a qualitative description was used. We conducted a qualitative analysis of 13 articles, which reported medication adherence based on different methods. Eight studies reported the medication adherence rate ranging from 20.0% to 92.2% (Charach et al., 2008; Hébert et al., 2013; Hodgkins et al., 2011; Ibrahim et al., 2002; Pelham et al., 2017; Perwien et al., 2004; Saloner et al., 2013; Sanchez et al., 2005). Five studies reported scores of self-report ratings or doctor ratings, ranging from 2.53 (1.73) out of four–8.86 (1.93) out of 10 (Firestone, 1982; Gimpel et al., 2005; Sleath et al., 2017; Sobanski et al., 2013; Zheng, 2015). Besides, the medication adherence was improved by economic incentives (Firestone, 1982) and decreased over time (Firestone, 1982; Sobanski et al., 2013) (more details in Supplementary Table S9).

### 3.5 Factors related to medication adherence

A total of 40 studies (63.5%) reported factors related to medication adherence for ADHD. Considering that the included intervention studies aimed to explore the improvement in adherence through intervention and could not reflect the influencing factors in a natural state. In this study, we only included 29 non-interventional studies, of which influencing factors were divided into five categories, including patient-related factors, disease-related factors, medication-related factors, caregiver-related factors, and environmental-related factors. The results of meta-analysis are shown in Table 5.

**TABLE 5:** Quantitative analysis of influencing factors.

Factors	N of study	N of patients	Effect model	Results of meta		Heterogeneity	
				OR	95%CI	*I* ^*2*^ (%)	*P*
Patient-related							
Genders	3	74,485	Random	1.07	1.03–1.11	89.20	<0.01
Medication-related							
Fewer medications	2	949	Random	0.45	0.27–0.75	0.00	<0.01
Parental approval of drug treatment	2	526	Random	1.87	1.16–3.02	91.00	0.01
Caregiver-related							
Parents had received medication education or training	3	1,304	Random	6.34	3.97–10.12	63.10	<0.01
Number of children in a family	2	1,167	Random	1.80	1.29–2.52	0.00	<0.01

OR, odds ratio.

#### 3.5.1 Patient-related factors

Seventeen studies reported patient-related factors, with three included in quantitative analysis and 13 included in qualitative analysis. The meta-analysis of three studies showed that the adherence of females was higher than that of males (Odds Ratio [OR] = 1.07, 95% CI:1.03–1.11, *I* ^*2*^ = 89.20%), consistent with three qualitative studies. Three studies suggest that the higher the self-awareness, intelligence, and emotional scores of children, the better adherence. Other factors were only reported in one study (Supplementary Table S10).

#### 3.5.2 Disease-related factors

Seven studies reported disease-related factors, with seven included in qualitative analysis. Three of the included studies reported the comorbidities, of which two studies found that the existence of comorbidities was associated with higher medication adherence. One study paid attention to ODD, and found that ODD was associated with poorer medication adherence. Other factors were only reported in a single study (Supplementary Table S10).

#### 3.5.3 Medication-related factors

Fifteen studies reported the medication-related factors, with four studies included in quantitative analysis and 11 in qualitative analysis. The meta-analysis indicated that increased frequency of medication use would reduce medication adherence (OR = 0.45, 95% CI:0.27–0.75, *I* ^*2*^ = 0.00%) and that parental approval of drug treatment would increase patients’ medication adherence (OR = 1.87, 95% CI:1.16–3.02, *I* ^*2*^ = 91.00%). Three studies showed that the adherence of sustained-release and controlled-release dosage forms was higher than that of immediate-release dosage forms. Two studies suggested that the side effects would decrease adherence. However, regarding the drug type, two studies have drawn opposite conclusions on whether stimulants would increase patients’ adherence. Other factors were only reported in a single study (Supplementary Table S10).

#### 3.5.4 Caregiver-related factors

Five studies reported the caregiver-related factors, with five studies included in quantitative analysis and three in qualitative analysis. The results of the meta-analysis indicated that children from single-child families had better adherence (OR = 1.80, 95% CI:1.29–2.52, *I* ^*2*^ = 0.00%), and that the adherence of children would be better if their parents had received medication education or training (OR = 6.34, 95% CI:3.97–10.12, *I* ^2^ = 63.10%). Two studies suggested that parental indulgence, parental negative emotions, and corporal punishment were risk factors for poor adherence. Other factors were reported in only a single study (Supplementary Table S10).

#### 3.5.5 Environment-related factors

Environment-related factors were only reported in one study. The study showed that the adherence rate of patients treated in general hospitals was higher, and the adherence in Central Europe was higher than that in East Asia (71.6% VS 55.5%) (Supplementary Table S10).

### 3.6 Publication bias and sensitivity analysis

The funnel plot was used to evaluate the publication bias of the included studies, and the results showed potential publication bias. But Egger’s test showed no significant publication bias (*P* = 0.19). There was no significant change before and after the trim and fill method (Supplementary Figure S6).

For the first sensitivity analysis, we excluded low-quality studies, and the meta-analysis results showed an adherence rate of 46.9% (95%CI 40.5%–53.3%; *I* ^*2*^ = 99.90%), with no significant changes observed (Supplementary Figure S7). For the second sensitivity analysis, we identified the top five studies that were most responsible for the observed publication bias by the Baujat plot, and repeated meta-analysis, publication bias assessment, and trim and fill method, and found no significant changes. For the third type of sensitivity analysis, the meta-analysis of adherence rates based on objective measures yielded a pooled estimate of 22.9% (95%CI 17.2%–29.2%, *I* ^*2*^ = 99.94%), whereas the estimate based on subjective measures was 49.1% (95%CI 25.0%–73.5%, *I* ^*2*^ = 98.01%). Compared with the main analysis, the objective adherence rate was lower, while the subjective rate was broadly consistent with the main analysis. This discrepancy is primarily attributable to the fact that the newly defined methods were predominantly subjective and contributed more data in the main analysis. For the forth type of sensitivity analysis, we found an adherence rate of 20.8% (95%CI 16.9%–25.0%, *I* ^*2*^ = 99.631%), which is also lower than the main analysis. We found that after applying stricter inclusion and exclusion criteria, the measurement methods for adherence shifted to being mainly objective, resulting in a lower adherence rate compared with main analysis but was close to the range observed in the third type of sensitivity analysis. Finally, although the four sensitivity analyses indicate relative robustness of the findings, the high heterogeneity warrants a cautious interpretation of these results.

## 4 Discussion

### 4.1 Statement of main findings and comparison with other studies

This systematic review evaluated the medication adherence rate and influencing factors for children and adolescents with ADHD.

Our study found the pooled medication adherence rate was 43.6% (95% CI, 39.0%–48.3%), slightly lower than a previous systematic review, which reported a 12-month stimulant adherence rate of 0.57 (Gajria et al., 2014). In the former systematic review, they only included two studies of children and adolescents with ADHD, which focused on stimulants, used MPR to measure adherence, and followed up for 12 months. However, in our study, we included 63 studies in total and 50 for quantitative analysis, which focused on both stimulants and non-stimulants, used more types of adherence measurements, and followed for a longer time. The difference in the included studies of these two systematic reviews may be related to the different results in the medication adherence rates of these two systematic reviews.

In addition, in the sensitivity analysis, we found that the adherence rate of subjective adherence measurements was higher than that of objective (subjective: 49.1%, objective: 22.9%). This pattern was even more pronounced in the forth sensitivity analysis, where objective measurements were mainly and yielded an adherence rate of 20.8%. A similar result was also observed in another study, where the subjective measurement indicated that patients missed 25% of their prescribed doses, while the objective measurement showed that they missed 40%–43%, suggesting a higher level of non-adherence as indicated by the objective measurement method (Schaefer et al., 2019). Although adherence rates from various measurement methods were illustrated in our subgroup and sensitivity analyses, most included studies utilized only a single approach. Consequently, the true adherence status could not be ascertained, making it impossible to determine the optimal adherence measurement tool for children with ADHD. The WHO states that no universally optimal medication adherence assessment method currently exists, advocating instead for a multi-method approach (WHO, 2003). Regarding method selection, Zullig LL et al. (Zullig et al., 2017) noted that choosing the optimal method requires a comprehensive consideration of the context, intended purpose, available resources, time, and phase of interest to make a final decision.

Regarding adherence-related factors, we explored five categories of factors, including patient-related, disease-related, medication-related, caregiver-related, and environmental-related factors. More than two studies reported the impact of 11 specific factors on adherence rate. Quantitative analysis revealed that female; parental agreement with the medication treatment plan; single-child families; and medication education or training for parents can improve medication adherence among children with ADHD, while higher medication doses could negatively impact adherence. Furthermore, qualitative evidence indicated that better intelligence and emotional status; using long-acting medications; and the presence of comorbidities are favorable factors, whereas older age; drug side effects; parental indulgence and negative emotions; and corporal punishment are adverse factors. However, there are still some inconsistencies. Khan et al. reported mixed effects of gender on adherence (Khan and Aslani, 2019), while our meta-analysis suggested that females had higher adherence. However, comorbidities and drug types are inconsistent in our study. For comorbidities, two studies reported higher medication adherence in children and adolescents with comorbidities, without reporting the specific type of comorbidities. However, if a patient’s comorbidity is ODD, their adherence may actually decrease, which may be because children with ODD are defiant and more likely to comply with adult requests to take medication (Thiruchelvam et al., 2001). For drug types, one included study reported higher adherence in stimulants, and another reported opposite results. A former systematic review indicated higher medication adherence in non-stimulants, while our meta-analysis of the rate of medication adherence indicated a higher adherence rate in stimulants (stimulants: 44.5%, non-stimulants: 24.1%). Stimulants, such as MPH, with some long-acting formulation, are a better choice for children and adolescents as the stigma associated with receiving medication during the school day (Hodgkins et al., 2012). Further studies are still needed to explore the impact of stimulants on medication adherence. Furthermore, previous studies on medication adherence in children or pediatric chronic diseases have also found that factors such as stigma, socioeconomic status, family support, route of administration, and medication palatability and so on may affect medication adherence in children (Hanghøj and Boisen, 2014; Walsh et al., 2014; Patel et al., 2015). Future studies could be conducted to explore the impact of these factors on medication adherence in children with ADHD.

### 4.2 Heterogeneity

Our study found high heterogeneity among medication adherence rates in children and adolescents with ADHD. The previous systematic review reported the same high heterogeneity, which performed qualitative analysis for most of the included studies and only conducted a meta-analysis on only two of them (Gajria et al., 2014). Another systematic review, which included adolescents and adults with asthma, also found a high heterogeneity of meta-analysis (I^2^ = 98.25%) (Zewdie et al., 2025). Similarly, another study including patients with cystic fibrosis only conducted qualitative analysis in their systematic review (Hansen et al., 2024). Thus, high heterogeneity is common across studies examining medication adherence rates.

In our study, Meta-regression indicated that study type, country, diagnosis, source of patients, formulation, and drug type may be potential causes of this heterogeneity. Firstly, in terms of study types, in addition to observational studies, this study also included the control groups of interventional studies, such as RCTs and non-RCTs, which were labeled as “intervention”. The results suggest that the adherence rate in the intervention subgroup is similar to that of cross-sectional studies but higher than that of cohort studies. This may be because the included cohort studies were primarily retrospective, while the intervention and cross-sectional studies often conducted surveys or follow-ups. In terms of sources of patients, the results showed that the adherence of patients recruited from EHR was lower than that from sources such as outpatient clinics or schools. Although EHR data has the advantage of larger sample sizes, potential issues with event capture may lead to an underestimation of the adherence rate (Hutchins et al., 2015). Furthermore, factors such as different countries, diagnostic criteria, drug formulations, and drug types (stimulants/non-stimulants) may also be potential sources of heterogeneity. In terms of countries and diagnostic criteria, variations in treatment protocols and initiation times can potentially influence adherence. Regarding drug formulations, we found significant differences in adherence between different drug formulations and types, which may contribute to heterogeneity. Besides, given the absence of a gold standard for measuring medication adherence, existing studies have utilized various measurement methods for assessment, part of our included studies used self-defined measurements, which may also be a potential source of the high heterogeneity. Regarding the measurement of adherence, there have been several guidelines on how to conduct research measuring adherence (De Geest et al., 2018; Gwadry-Sridhar et al., 2009; Peterson et al., 2007). However, none of these guidelines address issues such as selecting the most appropriate method for measuring adherence or determining the optimal follow-up period, which may be attributed to the fact that different diseases and populations require different adherence measurement methods. For instance, a study on adult cardiovascular diseases suggested that the Adherence to Refills and Medications Scale (ARMS) might be the optimal measurement tool (Tegegn et al., 2022), whereas another study on pediatric chronic diseases argued that electronic monitoring devices (e.g., MEMS) and pharmacy refill data are often more reliable than self-report questionnaires due to children’s limited self-reporting capabilities (Al-Hassany et al., 2019).

### 4.3 Quality of evidence

We found that cohort and cross-sectional studies were of moderate to high quality. However, there are still some limitations. Firstly, although missing data were common in cohort studies, several included studies did not report the missing data or the methods used to handle it. Secondly, the outcome evaluation did not apply blinding to the personnel involved in the evaluation, which may have led to bias. Lastly, in terms of the selection of study subjects, some studies did not report the selection of the control group. For RCTs, 57.1% of studies were rated as high-risk in terms of subject blinding, indicating that allocation concealment and blinding were important factors for the risk of RCT bias.

In this study, certain subgroup analyses were performed *post hoc* to investigate the sources of heterogeneity. Following the Cochrane Handbook guidelines (Deeks et al., 2024), we have clearly indicated in the text which subgroup analyses were preplanned or *post hoc*. However, as highlighted by Sun X et al. (Sun et al., 2010), the credibility of subgroup analyses heavily relies on whether they were preplanned and whether the expected direction of the subgroup effect was specified *a priori*. Because *post hoc* analyses can be data-driven, their validity might be affected. Therefore, the findings of our subgroup analyses must be interpreted with caution.

### 4.4 Limitation

Some limitations should be mentioned. Firstly, although we employed a random-effects model and conducted a meta-regression analysis, there is still high heterogeneity among the included studies. We have taken measurable methodological heterogeneity and clinical heterogeneity into consideration. However, in methodological heterogeneity, there may be other factors, for example, even among studies using MARS, variations in cut-off values and follow-up periods might have limited the observed reduction in heterogeneity. In clinical heterogeneity, there are some unmeasured factors, like comorbidities, treatment beliefs of children and their caregivers, and adverse drug events, that could not be extracted from the original studies. Thus, we could not access these impacts on heterogeneity. The meta-regression and part of subgroup analysis were *post hoc* analyisis, and we have conducted numerous subgroup analyses, which may increased risk of Type I error (false positives). Considering the high heterogeneity, all subgroup analyses and meta-regressions were conducted as exploratory analyses, and no adjustment for multiple comparison was applied, the results should be interpreted with caution, and more studies are needed in the future. Secondly, most of the included studies were cross-sectional studies and retrospective cohorts, which were inherently unable to provide follow-up or tracking, thus making it difficult to accurately reflect the relationship between medication adherence and follow-up time. Thirdly, the funnel plot suggested the potential publication bias, but the Egger’s test failed to find any publication bias, and the Trim and Fill method did not reveal significant differences in the results before and after adjustment. Even though no significant difference was found, there is still publication bias because the small-sample studies are less likely to be published in English. The results should be interpreted with caution. Fourth, although we have searched major databases, we did not conduct an exhaustive search of all databases such as PsychInfo. Consequently, some studies published exclusively in unsearched databases might have been missed, introducing potential selection bias. To mitigate this limitation, we supplemented our search with a grey literature search; however, no relevant grey literature was identified. Lastly, in terms of adherence-related factors, some studies that only described adherence-related factors without reporting adherence rates may have been excluded during the literature screening process.

### 4.5 Implications for clinical practice

Medication is still the primary treatment for ADHD, and studies have found that non-adherence with treatment can lead to adverse consequences such as declining academic performance, increased family and psychological stress, and increased risk of obesity and injuries (Abdallah et al., 2024). The results of our study indicate that medication adherence among children and adolescents with ADHD is still low, and is influenced not only by patient-related and disease-related factors but also by factors related to medications, caregivers, and environment. To improve medication adherence among these patients, several strategies can be implemented. Firstly, education for physicians is crucial to selecting appropriate medication prescriptions and minimizing potential adverse effects. Based on existing research, physicians should consider not only the efficacy and safety of medications but also the long-acting formulations that reduce the frequency of dosing, thereby encouraging patients to adhere to their medication. Secondly, since the adherence of children is influenced by their parents, it is essential to educate parents about the disease and medication (e.g., treatment characteristics, duration, dosage regimen, and administration methods) during consultations to correct any misconceptions they may have. Additionally, appropriate intervention measures can be adopted. Studies have found that interventions such as text message reminders, parents’ education and financial incentives can enhance medication adherence (Fried et al., 2020; Sobanski et al., 2013). However, high-quality RCTs are still needed to consolidate strategies for improving medication adherence in these patients.

### 4.6 Implications for future study

Our study found that almost 50% of studies used newly self-defined adherence measurement methods, and that the follow-up time and point vary in different studies, which may be associated with high heterogeneity of medication adherence. More unified measurement methods, guidelines for how to conduct medication adherence studies, and guidelines for how to report the results of medication adherence studies are urgently needed in future studies. Additionally, this study discovered that prolonged follow-up time may lead to decreased adherence, but existing research is unable to reflect the specific changes and reasons behind this. Recently, the group-based trajectory modeling (GBTM) method has been increasingly used in pharmacoepidemiological studies. GBTM could be used to synthesize adherence measurements across multiple time points, thereby describing medication adherence trajectories over time, and when combined with other quantitative analysis methods (e.g., logistic regression), can identify the reasons leading to different trajectories (Alhazami et al., 2020). Future studies can adopt the GBTM method to explore the dynamic trends of changes in adherence. Lastly, there is limited research on the factors influencing adherence, and some results are inconsistent. Further exploration is needed to understand the relationship between adherence and related factors, as well as potential mediating factors.

## 5 Conclusion

Improving medication adherence poses significant challenges for children and adolescents with ADHD. Although there are some consistent findings regarding certain influencing factors, there are still many inconsistent factors that necessitate further exploration. Furthermore, there is an urgent need to investigate strategies for improving medication adherence.

## Data Availability

The original contributions presented in the study are included in the article/Supplementary Material, further inquiries can be directed to the corresponding authors.
