# Supplementary material for: Medication adherence and influencing factors in children and adolescents with attention deficit hyperactivity disorder (ADHD): a systematic review and meta-analysis
Source: Front Pharmacol. 2026 Jul 23;17:1819206. doi: 10.3389/fphar.2026.1819206 (PMC13443319; doi:10.3389/fphar.2026.1819206)
Supplement: Supplementary file 1 [file Supplementaryfile1.docx]

**Supplementary Appendix 1. Detailed Search Strategy**

**PubMed**

#1 "Attention Deficit Disorder with Hyperactivity"[MeSH Terms] OR "ADHD"[title/abstract] OR "Attention Deficit Hyperactivity Disorder*"[title/abstract] OR "Attention Deficit Disorder with Hyperactivity"[title/abstract] OR " Attention Deficit Disorder*"[title/abstract] OR "Hyperkinetic Syndrome"[title/abstract]

#2 "Treatment Adherence and Compliance"[MeSH Terms] OR "Patient Compliance"[MeSH Terms] OR "Medication Adherence"[MeSH Terms] OR "compliance"[MeSH Terms] OR ((“medication"[title/abstract] OR “drug*"[title/abstract] OR “medicine*"[title/abstract] OR “treatment"[title/abstract] OR “therapeutic"[title/abstract]) AND (“compliance"[title/abstract] OR “adherence"[title/abstract] OR "persistence"[title/abstract]))

#3 "Adolescent"[Mesh] OR "Child"[Mesh] OR "Infant"[Mesh] OR adolescen*[Title/Abstract] OR babies[Title/Abstract] OR baby[Title/Abstract] OR boy?[Title/Abstract] OR boyfriend[Title/Abstract] OR boyhood[Title/Abstract] OR girlfriend[Title/Abstract] OR girlhood[Title/Abstract] OR child*[Title/Abstract] OR girl?[Title/Abstract] OR infan*[Title/Abstract] OR juvenil*[Title/Abstract] OR kid?[Title/Abstract] OR minors[Title/Abstract] OR minors*[Title/Abstract] OR neonat*[Title/Abstract] OR neo-nat*[Title/Abstract] OR newborn*[Title/Abstract] OR new-born*[Title/Abstract] OR paediatric*[Title/Abstract] OR peadiatric*[Title/Abstract] OR pediatric*[Title/Abstract] OR perinat*[Title/Abstract] OR preschool*[Title/Abstract] OR puber*[Title/Abstract] OR pubescen*[Title/Abstract] OR school*[Title/Abstract] OR teen*[Title/Abstract] OR toddler?[Title/Abstract] OR underage?[Title/Abstract] OR under-age?[Title/Abstract] OR youth*[Title/Abstract]

#4 #1 AND #2 AND #3

**Embase (Ovid)**

#1 exp attention deficit hyperactivity disorder/ or (ADHD or Attention Deficit Hyperactivity Disorder* or Attention Deficit Disorder with Hyperactivity or Attention Deficit Disorder* or Hyperkinetic Syndrome).ab,ti.

#2 exp medication compliance/ or exp patient compliance/ or exp "compliance (physical)"/ or ((medication or drug* or medicine* or treatment or therapeutic) and (compliance or adherence or persistence)).ab,ti.

#3 exp *adolescence/ or exp *adolescent/ or exp *child/ or exp *childhood disease/ or exp *infant disease/ or (adolescen* or babies or baby or boy? or boyfriend or boyhood or girlfriend or girlhood or child or child* or child*3 or children* or girl? or infan* or juvenil* or juvenile* or kid? or minors or minors* or neonat* or neo-nat* or newborn* or new-born* or paediatric* or peadiatric* or pediatric* or perinat* or preschool* or puber* or pubescen* or school* or teen* or toddler? or underage? or under-age? or youth*).ti,kw.

#1 AND #2 AND #3

**Cochrane Library**

#1 MeSH descriptor: [Attention Deficit Disorder with Hyperactivity] explode all trees or (ADHD or Attention Deficit Hyperactivity Disorder* or Attention Deficit Disorder with Hyperactivity or Attention Deficit Disorder* or Hyperkinetic Syndrome):ti,ab,kw

#2 MeSH descriptor: [Treatment Adherence and Compliance] explode all trees or MeSH descriptor: [Patient Compliance] explode all trees or MeSH descriptor: [Medication Adherence] explode all trees or MeSH descriptor: [Compliance] explode all trees or ((medication or drug* or medicine* or treatment or therapeutic) and (compliance or adherence or persistence)):ti,ab,k

#3 MeSH descriptor: [Adolescent] explode all trees or MeSH descriptor: [Child] explode all trees or MeSH descriptor: [Infant] explode all trees or (adolescen* or babies or baby or boy? or boyfriend or boyhood or girlfriend or girlhood or child or child* or child*3 or children* or girl? or infan* or juvenil* or juvenile* or kid? or minors or minors* or neonat* or neo-nat* or newborn* or new-born* or paediatric* or peadiatric* or pediatric* or perinat* or preschool* or puber* or pubescen* or school* or teen* or toddler? or underage? or under-age? or youth*):ti,ab,kw

**知网**

(题名或关键词 = ADHD OR 注意力缺陷多动障碍 OR 注意力缺陷多动症 OR 多动症 OR 注意力缺陷 OR 多动障碍 OR 多动综合征 OR 多动综合症) AND (题名或关键词 = 依从 OR 遵从 OR 顺从OR 服从 OR 持久) AND (题名或关键词 = 儿童 OR 青少年 OR 患儿 OR 小儿)

限定：模糊，同义词扩展，学术期刊+学位论文

限定时间，新增

**万方**

(题名或关键词 = ADHD OR 注意力缺陷多动障碍 OR 注意力缺陷多动症 OR 多动症 OR 注意力缺陷 OR 多动障碍 OR 多动综合征 OR 多动综合症) AND (题名或关键词 = 依从 OR 遵从 OR 顺从OR 服从 OR 持久) AND (题名或关键词 = 儿童 OR 青少年 OR 患儿 OR 小儿)

限定：模糊，主题词扩展，期刊论文+学位论文，语种：中文

**维普**

(题名或关键词 = ADHD OR 注意力缺陷多动障碍 OR 注意力缺陷多动症 OR 多动症 OR 注意力缺陷 OR 多动障碍 OR 多动综合征 OR 多动综合症) AND (题名或关键词 = 依从 OR 遵从 OR 顺从OR 服从 OR 持久) AND (题名或关键词 = 儿童 OR 青少年 OR 患儿 OR 小儿)

限定：模糊，同义词扩
