# Supplementary material for: Medication adherence and influencing factors in children and adolescents with attention deficit hyperactivity disorder (ADHD): a systematic review and meta-analysis
Source: Front Pharmacol. 2026 Jul 23;17:1819206. doi: 10.3389/fphar.2026.1819206 (PMC13443319; doi:10.3389/fphar.2026.1819206)
Supplement: Supplementary file 3 [file Supplementaryfile3.docx]

**Table S1** Details of types of interventions of RCTs and non-RCTs

| Author (Year) | Study Design | Intervention Group | Control Group |
| --- | --- | --- | --- |
| Xu XN. 2024(Xu et al., 2024) | RCT | Medication + Psychological Intervention | Medication + Parental Outpatient Education |
| Lv YB. 2023-1(Lv et al., 2023b) | RCT | Multimodal Comprehensive Intervention Group (Medication + Parent Training + Behavior Modification + Sensory Integration Therapy + Sandplay Therapy) | Medication |
| Lv YB. 2023-2(Lv et al., 2023a) | RCT | Pharmacological Treatment | Non-pharmacological Treatment |
| Huang YK 2020(Huang, Wang, Xie, & Wu, 2020) | RCT | Medication + Teacher Education | Medication |
| Zheng 2020(X. Zheng et al., 2020) | RCT | Medication + Parental Education + Teacher Education | Medication |
| Du X J 2019(Du et al., 2019) | RCT | Medication + Parental Group Training | Medication + Brief Parental Outpatient Education |
| Pelham 2017(Pelham et al., 2017) | Crossover | Medication | Placebo |
| Wang Y 2016(Y. Wang et al., 2016) | RCT | Medication + Teacher Education | Medication |
| Su 2016(Su, Yang, Stein, Cao, & Wang, 2016) | RCT | OROS MPH (Oral Osmotic Methylphenidate Hydrochloride) | ATX (Atomoxetine) |
| Zheng QM 2015a(Q. Zheng, 2015) | RCT | Medication + Shared Decision-Making (Doctor-Patient) | Medication + Doctor-Directed Decision-Making |
| Pappadopulos 2009(Pappadopulos et al., 2009) | RCT | Medication | NI (No Intervention) |
| Corkum 1999(Corkum, Rimer, & Schachar, 1999) | RCT | Medication + Parent Training | Placebo + Parent Training |
| Firestone 1982(Firestone, 1982) | RCT | Medication + Parent Training (Behavior Modification Guidance) | Medication |
| Fried 2020(Fried et al., 2020) | NRSI | Medication + SMS Reminders | Medication |
| Faraone 2007(Faraone, Biederman, & Zimmerman, 2007) | NRSI | Medication | NI (No Intervention) |

**Table S2 The definition of adherence in studies with newly self-defined adherence measurement methods.**

| **Author year** | **Study type** | **Classify** | **Definition of adherence** |
| --- | --- | --- | --- |
| Jia XH 2015 (X. Jia, 2015) | Cohort | Percentage of patients adhering to medication | Calculate the percentage of patients treated for 1, 3, 6 and ≥12 months out of the total number of patients followed up to evaluate treatment adherence |
| Chen GF 2011 (G. Chen, Cui, & Chen, 2011) | Cohort |  | Calculate the percentage of patients treated for 1, 3, 6 and ≥12 months out of the total number of patients followed up to evaluate treatment adherence |
| Lv YB 2023-2 (Lv et al., 2023b) | RCT | Adherence score | 3-point compliance scale: fully compliant, partially compliant, not compliant. |
| Shehadeh-Sheeny 2023 (Shehadeh-Sheeny & Baron-Epel, 2023) | Cross-section |  | 4-point adherence scale: 4 = very often, 1 = never. |
| Zheng QM 2015 (Q. Zheng, 2015) | RCT |  | Adherence to medication prescribed by the doctor was judged as good adherence to medication and was scored as 1, otherwise it was scored as 0. The total adherence score at the 4 follow-up visits (after 2, 4, 8 weeks and after 12 weeks) was an indicator out of 4. |
| Hong 2013 (J. Hong et al., 2013) | Cohort |  | Adherence score on a 5-point scale: 1 Never 2 Occasionally 3 Sometimes 4 Most of the time 5 Always. ≥4 for adherence, otherwise non-adherence. |
| Sobanski 2013 (Sobanski, Döpfner, Ose, & Fischer, 2013) | Cohort |  | 6-point adherence score: 1 = good adherence, 6 = inadequate adherence |
| Gimpel 2005 (Gimpel et al., 2005) | Cohort |  | Adherence score on a 10-point scale: 0 = never take medication, 5 = take medication about half the time it should be taken, 10 = always take medication when it should be taken |
| Sleath 2017 (Gimpel et al., 2005) | Cross-section |  | 1. 10 cm drawn line indicates how often all ADHD medications were used exactly as directed: 0 = no time, 10 = all time 2. child self-reported whether he/she did not take medication on non-school days (e.g., weekends or summer holidays) |
| Jia YL 2021 (Y. Jia et al., 2021) | Cross-section | Attendance at clinics on time | Timely administration of medication and regular review: telephone follow-up in the 2nd week of the child's first visit, 1 time/2 weeks. |
| Fried 2020 (Fried et al., 2020) | NRSI |  | Stimulant prescription defined as medication adherence within 37 days of receipt of text message in intervention group or registered letter in control group |
| Firestone 1982 (Firestone, 1982) | RCT | Adherence to medication duration | Adherence was defined as taking the drug for more than 9 consecutive months. |
| Atzori 2009 (Atzori, Usala, Carucci, Danjou, & Zuddas, 2009) | Cross-section |  | ≥80% of prescribed medications per month for ≥8 months per year as good adherence |
| Zheng 2020 (X. Zheng et al., 2020) | RCT |  | Good adherence was defined as 6 months of continuous regular medication. |
| Wang RF 2020 (R. Wang, Wei, Liu, Meng, & Liu, 2020) | Cross-section |  | Good adherence was defined as 6 months of continuous regular medication. |
| Du XJ 2019 (Du et al., 2019) | RCT |  | Good adherence was defined as 6 months of continuous regular medication, and the proportion of cases receiving medication was defined as the medication adherence rate. |
| Zhu DQ 2011 (Zhu, Hong, & Zhu, 2011) | Cross-section |  | Parents or caregivers of children with ADHD completed the study's self-developed Telephone Follow-up Questionnaire for Children with ADHD, and 6 months of continuous medication administration was defined as adherence. |
| Chen MR 2014 (M. Chen et al., 2014) | Cross-section |  | 6 months of continuous regular medication was defined as good adherence, the rest as poor |
| Wang Y 2011 (Y. Wang, Zhang, & Chen, 2011) | Cross-section |  | Self-developed parental questionnaire on medication adherence in children with ADHD, with 6 months of continuous regular medication use defined as good adherence. |
| Liu MZ 2011 (Liu, Chen, Lin, Chen, & Lv, 2011) | Cross-section |  | Self-administered medication adherence questionnaire, with good adherence defined as 3 months of continuous regular medication intake |
| Ding KJ 2013 (Ding et al., 2013) | Cross-section |  | Completion of 6 weeks of titrated therapy was defined as good compliance. |
| Saloner 2013 (Saloner, Fullerton, & McGuire, 2013) | Cohort | Percentage of days on medication | Annualised prescription days measure adherence |
| Sitholey 2011 (Sitholey, Agarwal, & Chamoli, 2011) | Cross-section |  | Adherence was defined as taking ≥80% of prescribed medications |
| Faraone 2007 (Faraone et al., 2007) | NRSI |  | Total days on medication divided by total study days ≥75% defined as good adherence |
| Charach 2004 (Charach, Ickowicz, & Schachar, 2004) | Cross-section |  | Children taking medication ≥5 days per week for more than 1 year during follow-up defined as adherence |
| Thiruchelvam 2001 (Thiruchelvam, Charach, & Schachar, 2001) | Cohort |  | Adherence was defined as a child taking medication ≥5 days per week during the follow-up period |
| Su 2016 (Su et al., 2016) | RCT |  | Adherence was defined as a child taking medication ≥5 days per week during the follow-up period |
| Hébert 2013 (Hébert, Polotskaia, Joober, & Grizenko, 2013) | Cross-section |  | Adherence measured by days of medication per week/7 days |
| Gau 2006 (Gau et al., 2006) | Cross-section |  | For the past 1 month, mothers reported missing any dose of IR MPH per day for more than 14 days defined as non-adherence |
| Kilic 2007 (Kiliç, Bilgiç, Gürkan, & Aysev, 2007) | Cohort | Number of medications taken | Cases in which weekly dosing exceeded 70% of the recommended dosage were considered adherent |
| Pelham 2017 (Pelham et al., 2017) | RCT(Cross over) |  | Percentage of doses taken as measured by pill counts and adherence calendars |
| Ibrahim 2002 (Ibrahim el, 2002) | Cross-section |  | Parents report adherence rates and verify by counting the number of pills remaining |
| Huang YK 2020 (Huang et al., 2020) | RCT | others | Children report taking medication at times and in doses exactly as prescribed by the doctor |

**Table S3 Characteristics of included studies**

| **Author year** | **Study type** | **Region** | **Sample size** | **Male** | **Age** | **Evaluation time** | **Diagnostic criteria** | **Comorbid** | **Participants** | **Exposure group** | **Control group** | **Definition** |
| --- | --- | --- | --- | --- | --- | --- | --- | --- | --- | --- | --- | --- |
| Earla 2022 (Earla, Abughosh, & Chen, 2022) | Cohort | America | 3083 | NI | 6-12 | 10M | ICD-9or ICD-10 | NI | EHR | NI | NA | PDC≥0.7 |
| Cheung 2021 (Cheung et al., 2021) | Cohort | Netherlands | 307 | 229 | 9.7(2.4) | 24M | NI | NI | EHR | MPH | NA | MPR≥80% |
| Ishizuya 2021 (Ishizuya et al., 2021) | Cohort | Japan | 1353 | 1141 | 9.65(2.85) | 12M | ICD-10 | NI | EHR | OROS-MPH | NA | MPR≥50% |
| Bhang 2017 (Bhang et al., 2017) | Cohort | Korean | 69631 | 53907 | 10.3(3.24) | NI | ICD-10 | NI | EHR | IR-MPH,ER-MPH and others^a^ | NA | MPR≥80% |
| Enns 2017 (Enns et al., 2017) | Cohort | Canada | 2369 | 2005 | NI | 5M | NI | NI | EHR | NI | AMPH, MPH and other^b^ | MPR≥80% |
| Bhang 2016 (Bhang et al., 2016) | Cohort | Korean | 10343 | 8223 | 6-18 | 36M | ICD-10 | NI | EHR | OROS-MPH, ER-MPH, ATX, IR-MPH | NA | MPR≥70% and MPR≥80% |
| Hong 2016 (M. Hong et al., 2016) | Cohort | Korean | 15133 | 11934 | 9.66(2.57) | 36M | ICD-10 | NI | EHR | IR-MPH, ER-MPH, OROS-MPH,ATX | NA | MPR≥60% and MPR≥70% and MPR≥80% |
| Jia XH 2015 (X. Jia, 2015) | Cohort | China | 132 | 78 | 5-13 | 12M | NI | NI | CD | IR-MPH | ER-MPH | Newly |
| Wehmeier 2015 (Wehmeier, Dittmann, & Banaschewski, 2015) | Cohort | German | 504 | 363 | 6-17 | 12M | ICD-10 or DSM-Ⅳ | CD, ODD, Anxiety, Depression, TD | CD | ATX | Stimulants | Other |
| Hong 2013 (J. Hong et al., 2013) | Cohort | Central Europe and East Asia | 860 | 702 | A,8.98(2.48),N,9.29 (2.67) | 12M | DSM-Ⅳ or ICD-10 | NI | CD | MPH and others | NA | Newly |
| Saloner 2013 (Saloner et al., 2013) | Cohort | America | 142625 | 72329 | 3-17 | 12M | ICD-9 | BD, Externalizing and others^c^ | CD | NI | NA | Newly |
| Setyawan 2013 (Setyawan et al., 2013) | Cohort | America | 101616 | NI | 6-17 | 12M | ICD-9 | Depressive, Anxiety, and others^k^ | EHR | LDX, ATX, OROS-MPH and others^d^ | LDX,ATX and other^e^ | PDC≥0.8 |
| Sobanski 2013 (Sobanski et al., 2013) | Cohort | German | 381 | NI | 14.0(1.9) | 70d | ICD-10 | LD, AS and others^f^ | CD | ER-MPH | NA | Newly |
| Tzang 2012 (Tzang et al., 2012) | Cohort | China TaiWan | 757 | 607 | 10.05(2.65) | 48W | DSM-Ⅳ | ODD | CD | IR-MPH, OROS-MPH, IR+OROS-MPH, Notreatment | NA | NI |
| Barner 2011 (Barner, Khoza, & Oladapo, 2011) | Cohort | America | 62789 | 43435 | 8.35(3.5) | NI | NI | Depression | EHR | Stimulants and non-stimulants | NA | MPR≥80% |
| Chen GF 2011 (G. Chen et al., 2011) | Cohort | China | 113 | 87 | 8.1(2.7) | 12M | DSM-Ⅳ | NI | CD | IR-MPH | SR-MPH | Newly |
| Hodgkins 2011-1 (Hodgkins, Sasané, & Meijer, 2011) | Cohort | America | 16799 | NI | 6-17 | 12M | ICD-9 | NI | EHR | MPH-LA | NA | MPR |
| Hodgkins 2011-2 (Hodgkins, Sasané, Christensen, Harley, & Liu, 2011) | Cohort | Netherlands | 4909 | 4030 | 6-17 | 12M | NI | NI | EHR | LA-MPH,IR-AMPH, and ATX | NA | MPR≥80% |
| Kilic 2007 (Kiliç et al., 2007) | Cohort | Turkey | 238 | 199 | 1A 11.61 ± 2.5,N,12.60 ± 2.5 | 12M | DSM-Ⅳ | ODD | CD | MPH | NA | Newly |
| Gimpel 2005 (Gimpel et al., 2005) | Cohort | America | 31 | 25 | NI | NI | NI | NI | CD | Stimulants | Non-medication | Newly |
| Sanchez 2005 (Sanchez, Crismon, Barner, Bettinger, & Wilson, 2005) | Cohort | America | 9549 | 7225 | 9.93(2.93) | 6M | NI | NI | CD | mixed AMPH, IR-MPH,OROS-MPH | NA | MPR |
| Perwien 2004 (Perwien, Hall, Swensen, & Swindle, 2004) | Cohort | America | 735 | 561 | 9.9(3.5) | 18M | ICD-9 | Depression | CD | MPH, D-AMPH, MDMA, or AMPH/D-AMPH | NA | MPR≥80% |
| Thiruchelvam 2001 (Thiruchelvam et al., 2001) | Cohort | Canada | 71 | NI | NI | 36M | DSM-III-R | NI | Trail | MPH, D-AMPH, and pemoline | NA | Newly |
| Alsubaie 2024 (Alsubaie et al., 2024) | Cross-section | Saudi Arabia | 221 | 201 | M7.90(2.40),F7.47(2.28) | NI | NI | LD | CD | AMPH,ATX and others^l^ | NA | MARS |
| Ragadran 2023 (Ragadran, Kamal Nor, Ismail, Ong, & Sundaraj, 2023) | Cross-section | Malaysia | 32 | NI | 6-17 | NI | Other | NI | CD | MPH, ATX, or risperidone | NA | MARS≥23 |
| Shehadeh-Sheeny 2023 (Shehadeh-Sheeny & Baron-Epel, 2023) | Cross-section | Israel | 18 | NI | NI | NI | DSM-III | NI | Schools | NI | NA | Newly |
| Nayak 2021 (Nayak et al., 2021) | Cross-section | India | 50 | 43 | 9.24(2.23) | NI | DSM-Ⅴ | ODD, CD, Depression, anxiety | Schools | ATX, MPH and risperidone | NA | Other |
| Jia YL 2021 (Y. Jia et al., 2021) | Cross-section | China | 50 | 42 | 10.5(1.5) | 2W | NI | NI | Schools | CR-MPH | NA | Newly |
| Wang RF 2020 (R. Wang et al., 2020) | Cross-section | China | 100 | 62 | 6-12 | 6M | NI | NI | CD | ER-MPH,ATX,IR-MPH | NA | Newly |
| Xie Feng 2020 (Xie et al., 2020) | Cross-section | China | 76 | 65 | 7.04(1.97) | 3M | DSM-Ⅳ | NI | CD | ATX | NA | Morisky≥8 |
| Safavi 2019 (Safavi, Saberzadeh, & Tehrani, 2019) | Cross-section | Iran | 118 | 98 | 8.89(1.9) | NI | DSM-Ⅴ | ODD | CD | ATX, MPH and other^g^ | NA | MARS≥23 |
| Emilsson 2017 (Emilsson, Gustafsson, Öhnström, & Marteinsdottir, 2017) | Cross-section | Sweden | 101 | 66 | 15.6(1.37) | NI | DSM-Ⅳ | NI | CD | MPH,ATX,ATX combined with MPH | NA | MARS≥23 |
| Sleath 2017 (Sleath et al., 2017) | Cross-section | America | 70 | 48 | 11.9(2.6) | NI | NI | NI | CD | NI | NA | Newly |
| Kosse 2017 (Kosse, Bouvy, Philbert, de Vries, & Koster, 2017) | Cross-section | Netherlands | 181 | 122 | 14.2(1.7) | NI | NI | NI | EHR | MPH and/or ATX | NA | MARS≥23 |
| Chen MR 2014 (M. Chen et al., 2014) | Cross-section | China | 490 | 447 | 8.6(1.8) | 6M | DSM-Ⅳ | NI | CD | MPH, ATX | NA | Newly |
| Ding KJ 2013 (Ding et al., 2013) | Cross-section | China | 188 | 165 | 9.4(2.2) | 6W | DSM-Ⅳ | ODD, CD | CD | IR-MPH | NA | Newly |
| Hébert 2013 (Hébert et al., 2013) | Cross-section | Canada | 33 | 26 | 8.78(1.77) | 3M | DSM-Ⅳ | NI | CD | SR-MPH and LDX | NA | Newly |
| Yang 2012 (Yang et al., 2012) | Cross-section | Korean | 39 | 35 | A10.11(2.17),N10.69(2.33) | 8W | DSM-Ⅳ | Depression, TD | CD | OROS-MPH | NA | Other |
| Liu MZ 2011 (Liu et al., 2011) | Cross-section | China | 310 | 223 | 9.8(2.7) | 3M | DSM-Ⅳ | NI | CD | NI | NA | Newly |
| Wang Y 2011 (Y. Wang et al., 2011) | Cross-section | China | 642 | 514 | 8.6(1.8) | 6M | DSM-Ⅳ | NI | CD | Stimulants | NA | Newly |
| Zhu DQ 2011 (Zhu et al., 2011) | Cross-section | China | 204 | 147 | 8.7(1.9) | 6M | DSM-Ⅳ | 133 of patients has comorbid | CD | MPH | NA | Newly |
| Sitholey 2011 (Sitholey et al., 2011) | Cross-section | India | 24 | 21 | 8.5(2.6) | NI | DSM-Ⅳ-TR | ODD,Enuresis, mental retardation | CD | IR-MPH or clonidine | NA | Newly |
| Atzori 2009 (Atzori et al., 2009) | Cross-section | Italy | 134 | 122 | 9(2) | 36M | DSM-Ⅳ | ODD/CD, mood and other^h^ | CD | SA-MPH | NA | Newly |
| Charach 2008 (Charach, Gajaria, Skyba, & Chen, 2008) | Cross-section | Canada | 19 | 16 | 11.9(2.1) | 3M | DSM-Ⅳ | NI | CD | Stimulants | NA | Other |
| Gau 2006 (Gau et al., 2006) | Cross-section | China Taiwan | 307 | 271 | 6-17 | 1M | NI | NI | CD | IR MPH | NA | Newly |
| Charach 2004 (Charach et al., 2004) | Cross-section | Canada | 79 | NI | NI | 60M | DSM-III-R | ODD | CD | Stimulants | NA | Newly |
| Ibrahim 2002 (Ibrahim el, 2002) | Cross-section | Saudi Arabia | 51 | 42 | 7-16.6 | 3M | DSM-Ⅳ or ICD-10 | NI | CD | MPH, imipramine and other^i^ | NA | Newly |
| Xu XN 2024 (Xu et al., 2024) | RCT | China | 124 | 68(I:33/62,C:35/62) | I:8.16(1.53),C:8.03(1.41) | NI | Other | NI | CD | ER-MPH ^m^ | ER-MPH | Mofisky-4 |
| Lv YB 2023-1 (Lv et al., 2023a) | RCT | China | 98 | 74(I:38 , C:36) | I:9.08(1.88),C:9.21(1.69) | NI | DSM-Ⅴ | NI | CD | Multi-modal integrated intervention group | ER-MPH, ATX | NI |
| Lv YB 2023-2 (Lv et al., 2023b) | RCT | China | 90 | 73 | I:9.03(1.78),C:9.03(1.78) | NI | DSM-Ⅴ | NI | CD | MPH, ATX | Nonmedication treatment | Newly |
| Huang YK 2020 (Huang et al., 2020) | RCT | China | 100 | 55(I:27/50,C:28/50) | 6-10 | 2M | Other | NI | CD | MPH + Medical Education Model Intervention | MPH | Newly |
| Zheng 2020 (X. Zheng et al., 2020) | RCT | China | 211 | NI | I:7.93(1.38),C:7.21(1.22) | 6M | DSM-Ⅴ | NI | Schools | MPH or ATX^n^ | MPH or ATX | Newly |
| Du XJ 2019 (Du et al., 2019) | RCT | China | 350 | 284(I:178/147,C:172/137 | 6-12 | 6M | DSM-Ⅳ | NI | CD | ER-MPH + Parent group training | ER-MPH | Newly |
| Pelham 2017 (Pelham et al., 2017) | RCT (Cross over) | America | 271 | 30 | 12-17  (M=14.1) | 12W | DSM-III | ODD | CD | MPH, placebo, and pemoline | NA | Newly |
| Wang Y 2016 (Y. Wang et al., 2016) | RCT | China | 170 | NI | NI | 6M | DSM-Ⅴ | NI | Schools | Medication ^o^ | Medication | NI |
| Su 2016 (Su et al., 2016) | RCT | China | 237 | NI | I:9.5(1.9), C:9.5(1.9) | 3M,6M,12M | DSM-Ⅳ | ODD | CD | OROS-MPH | ATX | Newly |
| Bai 2015 (Bai, Wang, Yang, & Niu, 2015) | RCT | China | 89 | 76(I:38/44,C:38/45) | I:9.3(2.8),C:9.6(2.9) | 1M,3M | DSM-Ⅳ | NI | CD | MPH or ATX ^p^ | MPH or ATX | MPR≥0.70 |
| Zheng QM 2015 (Q. Zheng, 2015) | RCT | China | 60 | NI | 6-12 | 12W | DSM-Ⅳ | NI | CD | Medication + Shared decision-making | Medication | Newly |
| Pappadopulos 2009 (Pappadopulos et al., 2009) | RCT | America | 254 | 201 | 7-9.9 | 14M | NI | NI | NI | IR-MPH | NA | Other |
| Johnston 1993 (Johnston & Fine, 1993) | RCT | Canada | 24 | NI | 6-10 | NI | DSM-III | NI | Mixed | MT：MPH^q^ | TCP：MPH^j^ | Other |
| Firestone 1982 (Firestone, 1982) | RCT | Canada | 76 | 68 | 5-9 | NI | NI | NI | CD | MPH + Behavior modification techniques | MPH | Newly |
| Fried 2020 (Fried et al., 2020) | NRSI | America | 333 | 245(I:65/87,C:180/246) | I:9.2(2.0), C:9.1(1.9) | NI | ICD-10 or DSM-Ⅴ | NI | CD | Stimulants + text message reminders | Stimulants | Newly |
| Faraone 2007 (Faraone et al., 2007) | NRSI | America | 407 | 338 | 9.2(1.8) | 12M | NI | ODD, CD | NI | OROS-MPH | NA | Newly |
| a, OROS-MPH,ATX, and combination  b, MAP, D-AMPH, LDX, ATX, pemoline and modafinil  c,Depression,Adjustment disorder,Asthma,Diabetes,Obesity  d,MPH-LA,MPH-SA,AMPH-LA,AMPH-SA  e, OROS-MPH,MPH-LA,MPH-SA,AMPH-LA,AMPH-SA  f, Speech and language disorders and,Depressive disorder, Anxiety, TD  g, , MPH combined with risperidone or clonidine  h, LD, TD, pervasive developmental disorder, Epilepsia, LD, Anxiety, Mental retardation  i,sertraline, clonidine, moclobemide  j, doses selected at the resident's discretion  k, Adjustment disorders  l, IR-MPH, ER-MPH, LDX,D-  m, Psychological intervention programme  n, Parent training and teacher training  o, Medical Education Model Intervention  p, well-designed psychoeducational programmes  q, （Fixed dose）, and compare with placebo  NI: No information  NA: Not applicable  Age: A:Adherence,N:Non-adherence,M:Male,F,Female  Study type：Cohort: cohort study, Cross-section: cross-section study, RCT: randomised controlled study, NRSI: non-randomised studies of the effects of interventions  Comorbid：ODD:oppositional defiant disorder,CD:conduct disorder, TD: Tic disorder/Tourette’s disorder, LD: learning disorder,BP:Bipolar disorder,AS:Asperger’s syndrome  Participants:CD: clinical department, EHR: electronic health records, Trial: clinical trail,  Drugs. AMPH:Amphetamine, ATX:Atomoxetine, D-AMPH: dextroamphetamine, LDX: Lisdexamfetamine,MAP: methamphetamine, MDMA:methamphetamine,MPH:methylphenidate, IR-MPH: Immediate-release methylphenidate, ER-MPH: extended-release,OROS-MPH: osmoticcontrolled release oral delivery system, MPH-LA:methylphenidate/dexmethylphenidate long acting, MPH-SA:methylphenidate/dexmethylphenidate short acting  Adherence. MPR:medication possession ratio, Newly: Newly defined methods | | | | | | | | | | | | |

**Table S4 Risk of bias of included cohort studies**

| **Study** | **Selection** | | | | **Comparability** | **Outcome** | | | **Quality Score** | **Result** |
| --- | --- | --- | --- | --- | --- | --- | --- | --- | --- | --- |
|  | **1)** | **2)** | **3)** | **4)** | **1)** | **1)** | **2)** | **3)** |  |  |
| Earla 2022 | 1 | 0 | 1 | 1 | 1 | 1 | 1 | 1 | 7 | High quality |
| Cheung 2021 | 1 | 1 | 1 | 1 | 2 | 1 | 1 | 1 | 9 | High quality |
| Ishizuya 2021 | 1 | 1 | 1 | 1 | 2 | 0 | 1 | 1 | 8 | High quality |
| Bhang 2017 | 1 | 1 | 1 | 1 | 2 | 1 | 0 | 1 | 8 | High quality |
| Enns 2017 | 1 | 0 | 1 | 1 | 2 | 0 | 1 | 1 | 7 | High quality |
| Hong 2016 | 1 | 1 | 1 | 1 | 2 | 1 | 1 | 1 | 9 | High quality |
| Bhang 2016 | 1 | 0 | 1 | 1 | 2 | 1 | 1 | 1 | 8 | High quality |
| Jia XH 2015 | 1 | 1 | 1 | 1 | 2 | 0 | 1 | 1 | 8 | High quality |
| Wehmeier 2015 | 1 | 0 | 1 | 1 | 2 | 0 | 1 | 0 | 6 | Medium quality |
| Saloner 2013 | 1 | 0 | 1 | 1 | 2 | 1 | 1 | 0 | 7 | High quality |
| Setyawan 2013 | 1 | 0 | 1 | 1 | 2 | 1 | 1 | 1 | 8 | High quality |
| Hong 2013 | 1 | 1 | 0 | 1 | 2 | 1 | 1 | 1 | 8 | High quality |
| Sobanski 2013 | 1 | 1 | 0 | 1 | 2 | 1 | 0 | 0 | 6 | Medium quality |
| Tzang 2012 | 1 | 0 | 1 | 1 | 2 | 0 | 1 | 0 | 6 | Medium quality |
| Barner 2011 | 1 | 0 | 1 | 1 | 2 | 1 | 0 | 0 | 6 | Medium quality |
| Chen GF 2011 | 1 | 1 | 1 | 1 | 2 | 0 | 1 | 1 | 8 | High quality |
| Hodgkins 2011-1 | 1 | 0 | 1 | 1 | 1 | 1 | 1 | 1 | 7 | High quality |
| Hodgkins 2011-2 | 1 | 0 | 1 | 1 | 2 | 1 | 1 | 0 | 7 | High quality |
| Kilic 2007 | 1 | 1 | 1 | 1 | 1 | 1 | 0 | 1 | 7 | High quality |
| Gimpel 2005 | 1 | 1 | 1 | 1 | 1 | 0 | 0 | 1 | 6 | Medium quality |
| Sanchez 2005 | 1 | 0 | 1 | 1 | 1 | 1 | 0 | 1 | 6 | Medium quality |
| Perwien 2004 | 1 | 0 | 1 | 1 | 1 | 1 | 1 | 0 | 6 | Medium quality |
| Thiruchelvam 2001 | 1 | 1 | 1 | 1 | 2 | 1 | 1 | 1 | 9 | High quality |

**Table S5	Risk of bias of included cross-sectional studies**

| **Study** | **Item 1** | **Item 2** | **Item 3** | **Item 4** | **Item 5** | **Item 6** | **Item 7** | **Item 8** | **Item 9** | **Item 10** | **Item 11** | **Quality Score** | **Result** |
| --- | --- | --- | --- | --- | --- | --- | --- | --- | --- | --- | --- | --- | --- |
| Alsubaie 2024 | Yes | Yes | Yes | Yes | Unclear | No | Yes | No | Yes | Yes | Yes | 8 | High quality |
| Ragadran 2023 | Yes | Yes | Yes | Yes | Unclear | Yes | Yes | No | Yes | Yes | Yes | 9 | High quality |
| Shehadeh-Sheeny 2023 | Yes | Yes | Yes | No | Unclear | Yes | Yes | No | Yes | Yes | Yes | 8 | High quality |
| Nayak 2021 | Yes | Yes | Yes | No | Unclear | Yes | Yes | Yes | Yes | Yes | Yes | 9 | High quality |
| Jia YL 2021 | Yes | No | Yes | No | Unclear | No | Yes | No | Yes | Yes | Yes | 6 | Medium quality |
| Wang RF 2020 | Yes | Yes | Yes | Unclear | Unclear | No | Yes | No | Yes | Yes | Yes | 7 | Medium quality |
| XIE Feng 2020 | Yes | Yes | Yes | Unclear | Unclear | No | Yes | Yes | No | Yes | Yes | 7 | Medium quality |
| Safavi 2019 | Yes | Yes | Yes | No | Unclear | No | Yes | Yes | Yes | Yes | Yes | 8 | High quality |
| Emilsson 2017 | Yes | Yes | Yes | Yes | Unclear | No | Yes | Yes | No | Yes | Yes | 8 | High quality |
| Sleath 2017 | Yes | Yes | No | No | Unclear | Yes | No | Yes | Yes | Yes | Yes | 7 | Medium quality |
| Kosse 2017 | Yes | Yes | No | No | Unclear | Yes | Yes | No | No | Yes | Yes | 6 | Medium quality |
| Chen MR 2014 | Yes | Yes | Yes | Yes | Unclear | No | Yes | Yes | No | Yes | Yes | 8 | High quality |
| Ding KJ 2013 | Yes | Yes | Yes | Unclear | Unclear | No | Yes | Yes | No | Yes | Yes | 7 | Medium quality |
| Hébert 2013 | Yes | Yes | Yes | No | Unclear | No | Yes | Yes | No | Yes | Yes | 7 | Medium quality |
| Yang 2012 | Yes | Yes | No | Yes | Unclear | Yes | Yes | Yes | No | Yes | Yes | 8 | High quality |
| Sitholey 2011 | Yes | Yes | No | No | Unclear | Yes | Yes | Yes | Yes | Yes | Yes | 8 | High quality |
| Zhu DQ 2011 | Yes | No | Yes | Yes | Unclear | No | Yes | Yes | No | Yes | Yes | 7 | Medium quality |
| Liu MZ 2011 | Yes | Yes | Yes | Yes | Unclear | No | Yes | No | No | Yes | Yes | 7 | Medium quality |
| Wang Y 2011 | Yes | Yes | Yes | Yes | Unclear | No | Yes | Yes | No | Yes | Yes | 8 | High quality |
| Atzori 2009 | Yes | Yes | Yes | Yes | Unclear | Yes | Yes | Yes | No | Yes | Yes | 9 | High quality |
| Charach 2008 | Yes | Yes | No | Unclear | No | Yes | Yes | Yes | No | Yes | Yes | 7 | Medium quality |
| Gau 2006 | Yes | Yes | No | Unclear | Unclear | No | Yes | Yes | No | Yes | Yes | 6 | Medium quality |
| Charach 2004 | Yes | Yes | No | No | Unclear | No | Yes | Yes | No | Yes | Yes | 6 | Medium quality |
| Ibrahim 2002 | Yes | Yes | Yes | No | Unclear | Yes | No | Yes | Yes | Yes | Yes | 8 | High quality |

**Table S6 Risk of bias of included RCTs**

| **Study** | **Random sequence generation (selection bias)** | **Allocation concealment (selection bias)** | **Blinding of participants and personnel (performance bias)** | **Blinding of outcome assessment (detection bias)** | **Incomplete outcome data (attrition bias)** | **Selective reporting (reporting bias)** | **Other bias** |
| --- | --- | --- | --- | --- | --- | --- | --- |
| Xu XN 2024 | Low risk | Unclear | Unclear | Unclear | Low risk | Low risk | Unclear |
| Lv YB 2023-1 | Low risk | Unclear | Unclear | Unclear | Low risk | Low risk | Unclear |
| Lv YB 2023-2 | Low risk | Low risk | High risk | Unclear | Low risk | Low risk | Unclear |
| Huang YK 2020 | Low risk | Unclear | High risk | Unclear | Low risk | Low risk | Unclear |
| Zheng 2020 | Low risk | Unclear | High risk | Unclear | Low risk | Low risk | Unclear |
| Du XJ 2019 | Low risk | Unclear | High risk | Unclear | Low risk | Low risk | Unclear |
| Wang Y 2016 | Unclear | Unclear | High risk | Unclear | Low risk | Low risk | Unclear |
| Su 2016 | Unclear | Unclear | High risk | Unclear | Low risk | Low risk | Unclear |
| Bai 2015 | Low risk | Unclear | High risk | Unclear | Low risk | Low risk | Unclear |
| Zheng QM 2015 | High risk | Unclear | Unclear | Unclear | Unclear | Low risk | Unclear |
| Pappadopulos 2009 | Unclear | Unclear | Low risk | Unclear | Low risk | Low risk | Unclear |
| Johnston 1993 | Unclear | Unclear | Unclear | Low risk | Unclear | Low risk | Unclear |
| Firestone 1982 | Unclear | Unclear | Unclear | Unclear | Unclear | Low risk | Unclear |
| Pelham 2017 | Unclear | Unclear | High risk | Unclear | Unclear | Low risk | Unclear |

**Table S7 Risk of bias of included NRSI**

| **Study** | **Pre-intervention** | | **At intervention** | **Post-intervention** | | | |
| --- | --- | --- | --- | --- | --- | --- | --- |
|  | **Bias due to confounding** | **Bias in selection of participants into the study** | **Bias in classification of interventions** | **Bias due to deviations from intended interventions** | **Bias due to missing data** | **Bias in measurement of outcomes** | **Bias in selection of the reported result** |
| Fried 2020 | Low risk | Low risk | Low risk | Low risk | High risk | Unclear | Unclear |
| Faraone 2007 | Low risk | Low risk | Low risk | Low risk | High risk | Low risk | Unclear |

**Table S8 More detailed information about studies included in quantitative analysis**

| Group | Subgroup | Median(%) | IQR(%) |  | Group | Subgroup | Median(%) | IQR(%) |
| --- | --- | --- | --- | --- | --- | --- | --- | --- |
| Study type | Cohort | 28.02 | 16.38-66.54 |  | Formulation | MPH | 28.79 | 14.60-62.17 |
|  | Cross-section | 52.63 | 38.44-62.13 |  |  | ER MPH | 60.00 | 30.48-60.00 |
|  | Intervention | 59.00 | 36.23-76.30 |  |  | IR MPH | 50.14 | 34.67-74.50 |
| Country | Canada | 52.77 | 43.67-77.38 |  |  | SR MPH | 80.96 | 72.92-92.15 |
|  | China | 60.00 | 37.30-74.34 |  |  | OROS MPH | 44.54 | 27.10-59.05 |
|  | Netherlands | 40.84 | 35.63-48.34 |  |  | AMPH | 13.34 | 10.07-16.64 |
|  | Korean | 26.32 | 19.33-29.87 |  |  | ATX | 20.99 | 13.43-31.88 |
|  | America | 16.64 | 10.51-21.38 |  |  | Others | 45.57 | 25.96-64.03 |
|  | Others | 63.52 | 53.92-78.73 |  | Drug type | Stimulants | 45.57 | 21.38-70.83 |
| Diagnosis | DSM-V | 49.42 | 35.02-68.83 |  |  | Non-stimulants | 20.99 | 13.43-31.88 |
|  | DSM-IV | 52.99 | 36.61-69.59 |  |  | Others | 46.53 | 22.98-64.53 |
|  | DSM-III-R | 52.38 | 44.30-59.92 |  | Follow time | 3M | 68.33 | 52.99-89.10 |
|  | ICD-10 | 26.32 | 19.33-29.87 |  |  | 6M | 39.88 | 35.63-60.00 |
|  | ICD-9 | 15.24 | 8.22-17.88 |  |  | 12M | 23.15 | 14.54-58.73 |
|  | Others | 67.49 | 22.98-74.27 |  |  | 24M | 38.51 | 21.18-56.54 |
| Source | Clinical department | 60.00 | 41.46-74.22 |  |  | 36M | 29.27 | 26.08-45.37 |
|  | EHR | 20.12 | 13.41-27.73 |  |  | Others | 52.13 | 21.24-74.96 |
|  | Others | 74.94 | 55.95-82.74 |  | Adherence | MARS | 49.83 | 34.30-67.04 |
| Drugs | MPH | 53.85 | 27.73-74.56 |  |  | MPR | 23.86 | 17.92-34.62 |
|  | AMPH | 13.34 | 10.07-16.64 |  |  | PDC | 14.95 | 9.93-17.81 |
|  | ATX | 20.99 | 13.43-31.88 |  |  | Newly defined methods | 60.00 | 39.57-74.49 |
|  | Others | 45.57 | 25.96-64.03 |  |  | Others | 67.36 | 59.96-76.30 |

**Table S9 Qualitative analysis of adherence rate**

| **Author year** | **Adherence Reporting and Definitions** | **Results** |
| --- | --- | --- |
| Saloner 2013 (Saloner et al., 2013) | Annualised Prescription Days (APD) | In 1996-1997，the APDs are highest for Whites(141 days), followed by Hispanics (106 days) and blacks (87 days)；In 2004-2005，the APDs are highest for Whites(184 days), followed by Hispanics (133 days) and blacks (121 days)； |
| Hébert 2013 (Hébert et al., 2013) | Days taken per week/7 days | Medication can be taken on time 83.7% of the time during the week |
| Hodgkins 2011-1 (Hodgkins, Sasané, & Meijer, 2011) Sanchez 2005 (Sanchez et al., 2005) Perwien 2004 (Perwien et al., 2004) | Mean value of MPR (SD) | MPR between 20.0% and 76.0% |
| Charach 2008 (Charach et al., 2008) Ibrahim 2002 (Ibrahim el, 2002) Pelham 2017(Pelham et al., 2017) | Number of tablets taken as a proportion of the total number of tablets taken | Percentage taking tablets between 56.3% and 92.2% |
| Gimpel 2005 (Gimpel et al., 2005) | Self-Report Scoring | Out of 10, the patient scored 8.86 (1.93) |
| Sleath 2017 (Sleath et al., 2017) | Self-Report Scoring | Out of 100 points, the average patient score was 77 points |
| Sleath 2017 (Sleath et al., 2017) | Self-reported if medication is not taken on non-school days | 74% (N=52) of children may not take their medication on non-school days, such as weekends and holidays. |
| Zheng QM 2015 (Q. Zheng, 2015) | Doctor's rating | Out of a possible 4 points, the average patient score was 2.53 (1.73) |
| Firestone 1982 (Firestone, 1982) | Doctor's rating | Adherence rates declined over time, with no significant differences between groups |
| Sobanski 2013 (Sobanski et al., 2013) | Doctor's rating | Financial incentives increased mean patient adherence by 0.7±1.2 points (p=0.001) |

**Table S10 Summary of factors associated with medication adherence**

|  |  | **Patient-related** | **Disease-related** | **Medication-related** | **Caregiver-related** | **Environment-related** |
| --- | --- | --- | --- | --- | --- | --- |
| Certain^*^ | Quantitative analysis | Female↑1.068(1.028,1.110)(N=3, n=74485) (Barner et al., 2011; Bhang et al., 2016; Ishizuya et al., 2021) |  | Frequency of medication↓ 0.447(0.266,0.752)  (N=2, n=949) (Gau et al., 2006; Y. Wang et al., 2011) | Parents had received medication education or training ↑6.341(3.974,10.120)  (N=3, n=1304) (M. Chen et al., 2014; Y. Wang et al., 2011; Zhu et al., 2011) |  |
|  |  |  |  | Parental approval of drug treatment 1.867(1.156,3.016)  (N=2, n=526) (M. Chen et al., 2014; Hébert et al., 2013) | Single-child families↑1.800(1.285-2.522)  (N=2, n=1167) (Cheung et al., 2021; J. Hong et al., 2013) |  |
|  | Qualitative analysis | Female↑  (N=2, n=167)  (Atzori et al., 2009; Hébert et al., 2013) |  | Sustained-release and controlled-release dosage forms↑  (N=3, n=132624) (Barner et al., 2011; Bhang et al., 2017; Zhu et al., 2011) |  |  |
|  |  | Self-awareness, intelligence, and emotional scores of children↑  (N=3, n=364) (Ding et al., 2013; R. Wang et al., 2020; Q. Zheng, 2015) |  | The side effects↓  (N=2, n=692) (Nayak et al., 2021; Y. Wang et al., 2011) |  |  |
|  |  | Age↓  (N=7, n=137911) (Atzori et al., 2009; Barner et al., 2011; Bhang et al., 2017; Gau et al., 2006; Hodgkins, Sasané, Christensen, et al., 2011; Sleath et al., 2017; Thiruchelvam et al., 2001) |  |  |  |  |
| Probable^#^ | Qualitative analysis |  | Comorbidities exists ↑  (N=2, n=69765)(Atzori et al., 2009; Bhang et al., 2017) ^&^ |  | Parental indulgence, parental negative emotions, and corporal punishment↓  (N=2, n=960) (J. Hong et al., 2013; R. Wang et al., 2020) |  |
| Uncertain^$^ | Qualitative analysis | Jewish > Arab  (N=1, n=18) (Shehadeh-Sheeny & Baron-Epel, 2023) | ODD↓(N=1, n=63)(Thiruchelvam et al., 2001) | Stimulants↑↓  (N=2, n=62839) (Barner et al., 2011; Nayak et al., 2021) | East Asia, ADHD Family History↓  (N=1, n=860) (J. Hong et al., 2013) | General hospitals ↑ (N=1,n=10343) (Bhang et al., 2016) |
|  |  | The white race↑  (N=1, n=70) (Sleath et al., 2017) | Poor concentration in class↓, children with mental retardation↑  (N=2, n=142) (Safavi et al., 2019; Sitholey et al., 2011) | Co-morbidity medication↑ sleeping pill combination↓  (N=2, n=64142) (Barner et al., 2011; Ishizuya et al., 2021) | Living with parents↓  (N=1, n=134) (Atzori et al., 2009) | Central Europe > East Asia (N=1, n=2) (J. Hong et al., 2013) |
|  |  |  | Distributed > Mixed; Combined and Hyperactive Impulsive > Attention Deficient (N=2, n=246) (Y. Wang et al., 2011; Q. Zheng, 2015) | Daily dose <18mg↓  (N=1, n=1353) (Ishizuya et al., 2021) |  |  |
|  |  |  | ADHD severity at baseline↓  (N=1, n=24) (Sitholey et al., 2011) | Long/short duration switching >2↓(N=1, n=307) (Cheung et al., 2021) |  |  |
| N: number of studies  n: number of patients  Certain^*^: Qualitative and quantitative studies have shown that the direction of influence of influencing factors on outcomes is consistently defined as Certain.  Probable^#^:Qualitative or quantitative research evidence only, with consistent direction defined as Probable.  Uncertain^$^:There were instances of inconsistencies in the direction of the included studies, or only a single study was defined as Uncertain.  And further classifying the influencing factors accordingly↑ Increased adherence, ↓ decreased adherence  &: we did not conduct a meta-analysis because one study reported OR and another reported HR. | | | | | | |

**Reference**

Alsubaie, M. A., Alshehri, Z. Y., Alawadh, I. A., Abulreesh, R. Y., Altaweel, H. M., & Alateeq, D. A. (2024). Treatment Adherence and Related Factors Among Children with Attention-Deficit/Hyperactivity Disorder in Saudi Arabia. *Patient Prefer Adherence, 18*, 337-348.

Atzori, P., Usala, T., Carucci, S., Danjou, F., & Zuddas, A. (2009). Predictive factors for persistent use and compliance of immediate-release methylphenidate: a 36-month naturalistic study. *J Child Adolesc Psychopharmacol, 19*(6), 673-681.

Bai, G. N., Wang, Y. F., Yang, L., & Niu, W. Y. (2015). Effectiveness of a focused, brief psychoeducation program for parents of ADHD children: improvement of medication adherence and symptoms. *Neuropsychiatr Dis Treat, 11*, 2721-2735.

Barner, J. C., Khoza, S., & Oladapo, A. (2011). ADHD medication use, adherence, persistence and cost among Texas Medicaid children. *Curr Med Res Opin, 27 Suppl 2*, 13-22.

Bhang, S. Y., Hwang, J. W., Kwak, Y. S., Joung, Y. S., Lee, S., Kim, B., et al. (2016). Differences in Utilization Patterns among Medications in Children and Adolescents with Attention-Deficit/Hyperactivity Disorder: a 36-Month Retrospective Study Using the Korean Health Insurance Review and Assessment Claims Database. *J Korean Med Sci, 31*(8), 1284-1291.

Bhang, S. Y., Kwack, Y. S., Joung, Y. S., Lee, S. I., Kim, B., Sohn, S. H., et al. (2017). Factors that Affect the Adherence to ADHD Medications during a Treatment Continuation Period in Children and Adolescents: A Nationwide Retrospective Cohort Study Using Korean Health Insurance Data from 2007 to 2011. *Psychiatry Investig, 14*(2), 158-165.

Charach, A., Gajaria, A., Skyba, A., & Chen, S. (2008). Documenting adherence to psychostimulants in children with ADHD. *J Can Acad Child Adolesc Psychiatry, 17*(3), 131-136.

Charach, A., Ickowicz, A., & Schachar, R. (2004). Stimulant treatment over five years: adherence, effectiveness, and adverse effects. *J Am Acad Child Adolesc Psychiatry, 43*(5), 559-567.

Chen, G., Cui, Y., & Chen, M. (2011). linical Effect of Control—-Released Methylphenidate Hydrochloride on Attention--Deficient Hyperactivity Disorder in Children. *Journal of Applied Clinical Pediatrics, 26*(6), 452-454,459.

Chen, M., Xue, Z., Huang, L., Chen, L., Huang, M., & Dong, X. (2014). Influencing factors of care-seeking and medication on children with attenation deficiit hyperactivity disorder. *Chinese Journal of Child Health Care, 22*(03), 315-318.

Cheung, K., Dierckx, B., El Marroun, H., Hillegers, M. H. J., Stricker, B. H., & Visser, L. E. (2021). Methylphenidate Treatment Adherence and Persistence in Children in the Netherlands. *J Child Adolesc Psychopharmacol, 31*(3), 205-213.

Corkum, P., Rimer, P., & Schachar, R. (1999). Parental knowledge of attention-deficit hyperactivity disorder and opinions of treatment options: impact on enrollment and adherence to a 12-month treatment trial. *Can J Psychiatry, 44*(10), 1043-1048.

Ding, K., Liu, Y., Kang, C., Liu, R., Li, X., & Wan, S. (2013). nfluential factors for methylphenidate treatment compliance in children with attention deficit hyperactivity disorder. *Chinese Journal of Contemporary Pediatrics, 15*(05), 343-347.

Du, X., Cheng, X., Zhang, W., Cheng, F., Hu, Z., Wang, B., et al. (2019). Effect of parental group training on drug therapy compliance in children with attention deficit hyperactivity disorder *Zhejiang Medical Journal, 41*(02), 154-156.

Earla, J. R., Abughosh, S., & Chen, H. (2022). Association of the Healthcare Effectiveness Data and Information Set (HEDIS) Follow-Up Care Measures and Medication Adherence Among Medicaid Insured Children with ADHD. *J Atten Disord, 26*(3), 370-376.

Emilsson, M., Gustafsson, P. A., Öhnström, G., & Marteinsdottir, I. (2017). Beliefs regarding medication and side effects influence treatment adherence in adolescents with attention deficit hyperactivity disorder. *Eur Child Adolesc Psychiatry, 26*(5), 559-571.

Enns, J. E., Randall, J. R., Smith, M., Chateau, D., Taylor, C., Brownell, M., et al. (2017). A Multimodal Intervention for Children with ADHD Reduces Inequity in Health and Education Outcomes. *Can J Psychiatry, 62*(6), 403-412.

Faraone, S. V., Biederman, J., & Zimmerman, B. (2007). An analysis of patient adherence to treatment during a 1-year, open-label study of OROS methylphenidate in children with ADHD. *J Atten Disord, 11*(2), 157-166.

Firestone, P. (1982). Factors associated with children's adherence to stimulant medication. *Am J Orthopsychiatry, 52*(3), 447-457.

Fried, R., DiSalvo, M., Kelberman, C., Adler, A., McCafferty, D., Woodworth, K. Y., et al. (2020). An innovative SMS intervention to improve adherence to stimulants in children with ADHD: Preliminary findings. *J Psychopharmacol, 34*(8), 883-890.

Gau, S. S., Shen, H. Y., Chou, M. C., Tang, C. S., Chiu, Y. N., & Gau, C. S. (2006). Determinants of adherence to methylphenidate and the impact of poor adherence on maternal and family measures. *J Child Adolesc Psychopharmacol, 16*(3), 286-297.

Gimpel, G. A., Collett, B. R., Veeder, M. A., Gifford, J. A., Sneddon, P., Bushman, B., et al. (2005). Effects of stimulant medication on cognitive performance of children with ADHD. *Clin Pediatr (Phila), 44*(5), 405-411.

Hébert, J., Polotskaia, A., Joober, R., & Grizenko, N. (2013). Adherence to psychostimulant medication in children with attention-deficit/hyperactivity disorder: the role of attitudes. *J Can Acad Child Adolesc Psychiatry, 22*(4), 317-323.

Hodgkins, P., Sasané, R., Christensen, L., Harley, C., & Liu, F. (2011). Treatment outcomes with methylphenidate formulations among patients with ADHD: retrospective claims analysis of a managed care population. *Curr Med Res Opin, 27 Suppl 2*, 53-62.

Hodgkins, P., Sasané, R., & Meijer, W. M. (2011). Pharmacologic treatment of attention-deficit/hyperactivity disorder in children: incidence, prevalence, and treatment patterns in the Netherlands. *Clin Ther, 33*(2), 188-203.

Hong, J., Novick, D., Treuer, T., Montgomery, W., Haynes, V. S., Wu, S., et al. (2013). Predictors and consequences of adherence to the treatment of pediatric patients with attention-deficit/hyperactivity disorder in Central Europe and East Asia. *Patient Prefer Adherence, 7*, 987-995.

Hong, M., Kim, B., Hwang, J. W., Bhang, S. Y., Choi, H. Y., Oh, I. H., et al. (2016). Naturalistic Pharmacotherapy Compliance among Pediatric Patients with Attention Deficit/Hyperactivity Disorder: a Study Based on Three-Year Nationwide Data. *J Korean Med Sci, 31*(4), 611-616.

Huang, Y., Wang, L., Xie, Y., & Wu, S. (2020). Application effect of free drug distribution combined with medical and educational model in children with attention deficit hyperactivity disorder. *CHINA MODERN MEDCINE, 27*(22), 107-110.

Ibrahim el, S. R. (2002). Rates of adherence to pharmacological treatment among children and adolescents with attention deficit hyperactivity disorder. *Hum Psychopharmacol, 17*(5), 225-231.

Ishizuya, A., Enomoto, M., Tachimori, H., Takahashi, H., Sugihara, G., Kitamura, S., et al. (2021). Risk factors for low adherence to methylphenidate treatment in pediatric patients with attention-deficit/hyperactivity disorder. *Sci Rep, 11*(1), 1707.

Jia, X. (2015). The Clinical Observation of Methylphenidate Hydrochloride Controlled Re-lease Tablets in the Treatment of Attention Deficit and Hyperactivity Disor-der in Children. *China & Foreign Medical Treatment, 34*(34), 147-148.

Jia, Y., Zhou, W., Li, Q., Jin, C., Zhi, H., & Chen, J. (2021). An investigation and analysis of adverse drug reactions and adherence in children taking methylphenidate hydrochloride controlled release for the treatment of ADHD. *Health Required*(7), 101-102.

Johnston, C., & Fine, S. (1993). Methods of evaluating methylphenidate in children with attention deficit hyperactivity disorder: acceptability, satisfaction, and compliance. *J Pediatr Psychol, 18*(6), 717-730.

Kiliç, B. G., Bilgiç, A., Gürkan, K., & Aysev, A. (2007). [Sociodemographic and clinical factors associated with compliance to methylphenidate treatment in children with attention deficit hyperactivity disorder]. *Turk Psikiyatri Derg, 18*(3), 207-213.

Kosse, R. C., Bouvy, M. L., Philbert, D., de Vries, T. W., & Koster, E. S. (2017). Attention-Deficit/Hyperactivity Disorder Medication Use in Adolescents: The Patient's Perspective. *J Adolesc Health, 61*(5), 619-625.

Liu, M., Chen, Y., Lin, G., Chen, J., & Lv, H. (2011). Analysis of factors affecting adherence to medication in children with attention deficit hyperactivity disorder. *Chinese Community Doctors, 13*(14), 20-21.

Lv, Y. B., Cheng, W., Wang, M. H., Wang, X. M., Hu, Y. L., & Lv, L. Q. (2023a). Effect of non-pharmacological treatment on the full recovery of social functioning in patients with attention deficit hyperactivity disorder. *World J Clin Cases, 11*(14), 3238-3247.

Lv, Y. B., Cheng, W., Wang, M. H., Wang, X. M., Hu, Y. L., & Lv, L. Q. (2023b). Multimodal integrated intervention for children with attention-deficit/hyperactivity disorder. *World J Clin Cases, 11*(18), 4267-4276.

Nayak, A. S., Nachane, H. B., Keshari, P., Parkar, S. R., Saurabh, K. H., & Arora, M. (2021). Prescription patterns and medication adherence in preadolescent children with attention deficit hyperactivity disorder. *Indian J Psychiatry, 63*(3), 274-278.

Pappadopulos, E., Jensen, P. S., Chait, A. R., Arnold, L. E., Swanson, J. M., Greenhill, L. L., et al. (2009). Medication adherence in the MTA: saliva methylphenidate samples versus parent report and mediating effect of concomitant behavioral treatment. *J Am Acad Child Adolesc Psychiatry, 48*(5), 501-510.

Pelham, W. E., Smith, B. H., Evans, S. W., Bukstein, O., Gnagy, E. M., Greiner, A. R., et al. (2017). The Effectiveness of Short- and Long-Acting Stimulant Medications for Adolescents With ADHD in a Naturalistic Secondary School Setting. *J Atten Disord, 21*(1), 40-45.

Perwien, A., Hall, J., Swensen, A., & Swindle, R. (2004). Stimulant treatment patterns and compliance in children and adults with newly treated attention-deficit/hyperactivity disorder. *J Manag Care Pharm, 10*(2), 122-129.

Ragadran, J., Kamal Nor, N., Ismail, J., Ong, J. J., & Sundaraj, C. (2023). Estimating the Risk of Attention Deficit Hyperactivity Disorder (ADHD) in Parents of Children with ADHD and the Association with Their Children's Disease Severity and Adherence to Medication. *Children (Basel), 10*(9).

Safavi, P., Saberzadeh, M., & Tehrani, A. M. (2019). Factors Associated with Treatment Adherence in Children with Attention Deficit Hyperactivity Disorder. *Indian J Psychol Med, 41*(3), 252-257.

Saloner, B., Fullerton, C., & McGuire, T. (2013). The impact of long-acting medications on attention-deficit/hyperactivity disorder treatment disparities. *J Child Adolesc Psychopharmacol, 23*(6), 401-409.

Sanchez, R. J., Crismon, M. L., Barner, J. C., Bettinger, T., & Wilson, J. P. (2005). Assessment of adherence measures with different stimulants among children and adolescents. *Pharmacotherapy, 25*(7), 909-917.

Setyawan, J., Hodgkins, P., Guérin, A., Gauthier, G., Cloutier, M., Wu, E. Q., et al. (2013). Comparing treatment adherence of lisdexamfetamine and other medications for the treatment of attention deficit/hyperactivity disorder: a retrospective analysis. *J Med Econ, 16*(7), 962-975.

Shehadeh-Sheeny, A., & Baron-Epel, O. (2023). Prevalence, diagnosis and treatment of ADHD in Arab and Jewish children in Israel, where are the gaps? *BMC Psychiatry, 23*(1), 586.

Sitholey, P., Agarwal, V., & Chamoli, S. (2011). A preliminary study of factors affecting adherence to medication in clinic children with attention-deficit/hyperactivity disorder. *Indian J Psychiatry, 53*(1), 41-44.

Sleath, B., Carpenter, D. M., Sayner, R., Thomas, K., Mann, L., Sage, A., et al. (2017). Youth Views on Communication About ADHD and Medication Adherence. *Community Ment Health J, 53*(4), 438-444.

Sobanski, E., Döpfner, M., Ose, C., & Fischer, R. (2013). A non-interventional study of extended-release methylphenidate in the routine treatment of adolescents with ADHD: effectiveness, safety and adherence to treatment. *Atten Defic Hyperact Disord, 5*(4), 387-395.

Su, Y., Yang, L., Stein, M. A., Cao, Q., & Wang, Y. (2016). Osmotic Release Oral System Methylphenidate Versus Atomoxetine for the Treatment of Attention-Deficit/Hyperactivity Disorder in Chinese Youth: 8-Week Comparative Efficacy and 1-Year Follow-Up. *J Child Adolesc Psychopharmacol, 26*(4), 362-371.

Thiruchelvam, D., Charach, A., & Schachar, R. J. (2001). Moderators and mediators of long-term adherence to stimulant treatment in children with ADHD. *J Am Acad Child Adolesc Psychiatry, 40*(8), 922-928.

Tzang, R. F., Wang, Y. C., Yeh, C. B., Hsu, C. D., Liang, H. Y., Yang, P. C., et al. (2012). Naturalistic exploration of the effect of osmotic release oral system-methylphenidate on remission rate and functional improvement in Taiwanese children with attention-deficit-hyperactivity disorder. *Psychiatry Clin Neurosci, 66*(1), 53-63.

Wang, R., Wei, G., Liu, X., Meng, Z., & Liu, Y. (2020). A study of factors influencing medication adherence in children with attention deficit hyperactivity disorder. *SOUTH CHINA JOURNAL OF PREVENTIVE MEDICINE, 46*(04), 409-411.

Wang, Y., Wei, S., Chen, J., Jiang, L., He, L., Zheng, X., et al. (2016). Study on nedicine combine with education in primary students with attention-deficit hyperactivity disorder in Jiangan and Putuo district of Shanghai. *Chinese Journal of Child Health Care | Chin J Child Heal Care, 24*(06), 587-589+600.

Wang, Y., Zhang, F., & Chen, J. (2011). Study on factors affecting medication compliance in children with attention deficit hyperactivity disorder. *Chinese Journal of Child Health Care | Chin J Child Heal Care, 19*(10), 946-948.

Wehmeier, P. M., Dittmann, R. W., & Banaschewski, T. (2015). Treatment compliance or medication adherence in children and adolescents on ADHD medication in clinical practice: results from the COMPLY observational study. *Atten Defic Hyperact Disord, 7*(2), 165-174.

Xie, F., Liu, X., LIin, H., Xiao, P., Zheng, X., Huang, Y., et al. (2020). Efficacy and safety of tomoxetine hydrochloride in children with attentiondeficit/hyperactivity disorder. *Central South Pharmacy, 18*(11), 1920-1927.

Xu, X., Zhang, J., Huang, J., Suo, Y., Cui, C., & Wng, C. (2024). Psycho-behavioural interventions, parent group training in the treatment of children with attention deficit hyperactivity disorder. *International Journal of Nursing*.

Yang, J., Yoon, B. M., Lee, M. S., Joe, S. H., Jung, I. K., & Kim, S. H. (2012). Adherence with electronic monitoring and symptoms in children with attention deficit hyperactivity disorder. *Psychiatry Investig, 9*(3), 263-268.

Zheng, Q. (2015). *Impacts of Shared Decision-Making and Other Factors on the Medication Compliance ofADHD Patients.* Unpublished 硕士.

Zheng, X., Shen, L., Jiang, L., Shen, X., Xu, Y., Yu, G., et al. (2020). Parent and Teacher Training Increases Medication Adherence for Primary School Children With Attention-Deficit/Hyperactivity Disorder. *Front Pediatr, 8*, 486353.

Zhu, D., Hong, H., & Zhu, Y.-y. (2011). Investigation of the compliance of attention deficient hyperactivity disorder children on methylphenidate therap. *Chinese Journal of Child Health Care | Chin J Child Heal Care, 19*(03), 267-269.
